# Loss of Alkbh5 enhances AT2 cell differentiation and alveolar repair across diverse injury models via m^6^A-dependent Areg signaling

**DOI:** 10.7150/ijbs.130452

**Published:** 2026-06-25

**Authors:** Jie Lv, Kechen Chen, Xinlong Wang, Yuchen Liu, Lian Li, Shijun Chen, Weiqi Lv, Yanqun Zhang, Yonghao Xu, Qian Liu, Guodong Hu, Zhili Rong, Xin Zhang, Ying Lin

**Affiliations:** 1 Cancer Research Institute, School of Basic Medical Sciences, State Key Laboratory of Multi-organ Injury Prevention and Treatment, Guangdong Province Key Laboratory of Immune Regulation and Immunotherapy, Southern Medical University, Guangzhou 510515, China.; 2 Huizhou Central People's Hospital Postdoctoral Innovation Practice Base, Southern Medical University, Huizhou 516008, China.; 3 Department of Critical Care Medicine, the First Affiliated Hospital of Guangzhou Medical University, Guangzhou 530021, China.; 4 School of Biomedical Engineering, Guangzhou Medical University, Guangzhou, Guangdong, 511436, China.; 5 Department of Detection and Diagnosis Technology Research, Guangzhou National Laboratory, Guangzhou, Guangdong, 510000, China.; 6 Department of Respiratory and Critical Care Medicine, Institute of Respiratory and Critical Care Medicine, The Tenth Affiliated Hospital of Southern Medical University (Dongguan People's Hospital), Dongguan 523000, China.; 7 State Key Laboratory of Respiratory Disease, National Clinical Research Center for Respiratory Disease, National Center for Respiratory Medicine, Guangzhou Institute of Respiratory Health, the First Affiliated Hospital of Guangzhou Medical University, Guangzhou 510091, China.

**Keywords:** alveolar regeneration, AT2, m^6^A RNA modification, Alkbh5, Areg

## Abstract

Efficient regeneration of the alveolar epithelium is essential for restoring lung function after injury, yet the mechanisms that govern alveolar type II cell (AT2) behavior remain insufficiently defined. Here, we identify the m⁶A RNA demethylase Alkbh5 as a pivotal regulator of AT2 cell activation and lineage progression. Conditional deletion of Alkbh5 in AT2 cells markedly enhances their proliferation and differentiation across diverse lung injury contexts—including fibrotic (bleomycin), inflammatory (LPS), mechanical (pneumonectomy), and oxidative (BHT) insults. Loss of Alkbh5 increases m⁶A modification on Amphiregulin (Areg) transcripts, stabilizing its mRNA and elevating Areg expression specifically within transitional AT2 populations. The resulting amplification of EGFR signaling drives accelerated AT2-to-AT1 differentiation and epithelial repair. Supplementation of recombinant Areg phenocopies the regenerative effects of Alkbh5 deletion, whereas Areg neutralization abrogates these responses, establishing Areg as a key downstream effector of Alkbh5. Importantly, ALKBH5 ablation or pharmacologic inhibition in human ESC-derived alveolar organoids similarly promote proliferation, differentiation, and AREG upregulation, demonstrating evolutionary conservation of this regulatory axis. Together, our findings reveal an Alkbh5-Areg-EGFR circuit that orchestrates alveolar epithelial regeneration and suggest new therapeutic opportunities for enhancing lung repair following injury.

## Introduction

The lung is a vital organ responsible for gas exchange, ensuring oxygen uptake into the bloodstream and the removal of carbon dioxide. Alveoli, the fundamental units of pulmonary gas exchange, are primarily composed of two epithelial cell types: alveolar type I (AT1) and type II (AT2) cells^1^. AT1 cells facilitate gas exchange, whereas AT2 cells synthesize and secrete pulmonary surfactant to maintain alveolar surface tension and prevent collapse. The alveolar epithelium lines the internal surface of the alveoli and is directly exposed to environmental insults, including airborne toxins, irritants, and pathogens. To preserve respiratory function, the alveolar epithelium requires continuous homeostatic maintenance and efficient regeneration following injury^2^. After alveolar injury, multiple stem and progenitor cell populations contribute to lung regeneration. Bronchioalveolar stem cells (BASCs), located at the bronchioalveolar duct junction, represent a multipotent cell population that supports both bronchiolar repair and alveolar regeneration by differentiating into AT2 cells 3, 4. Similarly, Club cells residing in the airway epithelium can transdifferentiate into AT2 cells following injury, contributing to alveolar reconstruction 5, 6. Earlier studies proposed that AT1 cells may act as progenitors during lung repair; however, more recent lineage-tracing analyses have demonstrated that AT1 cells are terminally differentiated and do not significantly contribute to alveolar regeneration^6^. AT2 cells are widely recognized as the principal stem/progenitor population that self-renews and differentiates into AT1 cells under both homeostatic and injury conditions 7, 8.

N6-methyladenosine (m^6^A) is one of the most prevalent internal modifications in eukaryotic mRNA. m^6^A modifications are broadly distributed across various RNA species, including mRNA, tRNA, rRNA, and long non-coding RNAs (lncRNAs)^9^, ^10^. The modification typically occurs within a conserved RRACH consensus motif (where R = A or G; H = A, C, or U) 11. By regulating multiple aspects of RNA metabolism—including RNA stability, splicing, nuclear export, and translation—m^6^A plays a critical role in modulating gene expression, protein function, chromatin structure, and histone modification^12^-^14^. Dysregulation of m^6^A has been implicated in the pathogenesis of various diseases^12^, ^15^, ^16^. However, while most studies on m^6^A in the lung have focused on cancer^17^-^19^, its role in alveolar regeneration remains poorly understood^20^, ^21^. Despite emerging evidence linking m^6^A regulation to lung injury responses, how m^6^A dynamics shape alveolar regeneration is unknown. To address this question, we examined the m^6^A demethylase Alkbh5, whose selective deletion in AT2 cells provides a tractable system for probing epitranscriptomic control of epithelial repair.

Amphiregulin (Areg) is a member of the epidermal growth factor (EGF) family that functions through autocrine and paracrine signaling in various cell types, including epithelial cells, immune cells, and cancer cells^22^-^25^. Areg exerts its biological effects by activating the epidermal growth factor receptor (EGFR), thereby promoting cell proliferation and differentiation—functions particularly crucial for tissue repair and regeneration^26^. Upon epithelial injury, Areg expression is markedly upregulated, facilitating rapid recovery of damaged tissues. In addition to promoting epithelial proliferation, Areg influences cell fate decisions, accelerating the regenerative process^26^, ^27^. As a non-selective EGFR ligand, Areg activates EGFR signaling in multiple cell types, including epithelial stem/progenitor cells and fibroblasts. In epithelial stem cells, Areg-mediated EGFR activation enhances tissue remodeling and repair, essential processes for wound healing and organ regeneration^26^, ^28^-^30^. In the lung, regulatory T cells (Tregs) have been shown to secrete Areg, activating fibroblasts and stimulating fibroblast growth factor (Fgf7 and Fgf10) production to promote lung regeneration^31^. However, the role of Areg in directly regulating AT2 cell function has not been well characterized.

To address these gaps, we investigated the role of the m^6^A demethylase Alkbh5 in regulating AT2-mediated alveolar regeneration. Through a combination of genetic models, organoid cultures, and multi-omics analyses, we uncovered a conserved Alkbh5-Areg-EGFR signaling axis that controls AT2 cell activation and accelerates lung repair after injury.

## Materials and Methods

### Animals

All animal experiments in this study were conducted in accordance with protocols approved by the Institutional Animal Care and Use Committee of Southern Medical University. Mice were housed in a temperature- and humidity-controlled facility under a 12-hour light/12-hour dark cycle at a constant temperature of 23°C, with ad libitum access to food and water.

### Ethics statement

Our research complies with all relevant ethical regulations, and mice experiments were approved by the Institutional Animal Care and Use Committee (IACUC) of Southern Medical University (Number: 2019023).

All transgenic mouse strains used in this study were maintained on a C57BL/6 genetic background. Alkbh5^flox/flox^ mice^32^ were kindly provided by Prof. Xiong Cao at Southern Medical University, Rosa26-mTmG were kindly provided by Prof. Weijie Zhou at Southern Medical University and Rosa26-Brainbow mice were generously provided by Prof. Meng Zhao at Sun Yat-sen University. Sftpc-CreERT2 mice were purchased from Jackson Laboratory (Stock No. 028054). Areg^flox/flox^ mice (Strain S-CKO-17789) were purchased from Cyagen. Genotyping was performed by PCR, and the primer sequences are listed in the Supplementary Table 1. In the experiments, Alkbh5^flox/flox^; Sftpc-CreERT2; Rosa26-mTmG mice were used as the conditional knockout group, while Alkbh5^flox/+^; Sftpc-CreERT2; Rosa26-mTmG littermates served as controls.

### Method details

#### Animal models

Tamoxifen was administered to mice at a dose of 200 mg/kg by intraperitoneal injection every other day for a total of four injections to induce gene deletion or lineage labeling. One week after the final injection, mice were subjected to subsequent injury models. For cell-sorting experiments, mice received tamoxifen intraperitoneally at 200 mg/kg daily for 3 consecutive days, and experiments were performed 4 days after the final injection.

#### BHT-induced lung injury

Butylated hydroxytoluene (BHT, Sigma; B1378) was dissolved in corn oil at a concentration of 30 mg/mL. One week after tamoxifen administration, conditional knockout and control mice were intraperitoneally injected with BHT at a dose of 225 mg/kg. Lung tissues were harvested on day 7 and day 14 post-injection for further analysis.

#### Bleomycin-induced lung injury

One week after tamoxifen-induced gene deletion, conditional knockout and control mice were anesthetized with isoflurane, and bleomycin (3 U/kg, TCI; B3972) dissolved in sterile NaCl solution was administered via intratracheal aerosolization using a microsprayer. Lung tissues were collected on days 14 and 21, as well as at one year after bleomycin administration, for subsequent analysis.

#### LPS-Induced lung injury

One week following tamoxifen treatment, mice were anesthetized with isoflurane. Lipopolysaccharide (LPS, 250 μg/kg, Sigma; L2360) diluted in 80 μL phosphate-buffered saline (PBS) was delivered intratracheally using a microsprayer (YUYAN, YAN-30012). Lung samples were harvested on days 7 and 14 post-injury.

#### Pneumonectomy (PNX) model

One week after tamoxifen induction, *mice* were anesthetized with tribromoethanol. After intubation and mechanical ventilation, a left thoracotomy was performed to expose the left lung. The left lung lobe was resected and the bronchial stump ligated. The thoracic cavity was closed, and mice were allowed to recover. Lungs were collected on days 7 and 14 post-PNX for analysis.

#### Preparation of single-cell suspensions from mouse lungs

Mice were euthanized by overdose of tribromoethanol. After opening the thoracic cavity, the trachea was exposed and isolated. Mice were perfused through the right ventricle with 5 mL of PBS after incision of the left atrium, until the lungs turned pale. Subsequently, 1.5 mL of digestion buffer (Dispase II, 5 mg/mL; Collagenase I, 1 mg/mL; both in HBSS) was gently instilled into the lungs via the trachea, which was then ligated immediately to retain the enzyme solution.

The lungs, along with the trachea, were transferred into a 15 mL centrifuge tube and incubated at room temperature for 45 minutes. After digestion, the trachea was removed, and the remaining lung tissue was minced into ~2-3 mm³ fragments in a 6-cm dish. The tissue fragments were dissociated by pipetting with a cut 1 mL tip, followed by an additional 10-minute digestion. Enzymatic activity was terminated by adding DMEM containing 10% fetal bovine serum (FBS). The suspension was passed through a 70 μm cell strainer and centrifuged at 200 × g for 15 minutes at 4°C. The resulting pellet was resuspended in 4 mL red blood cell (RBC) lysis buffer and incubated briefly. After RBC lysis, 4 mL DMEM was added, followed by another round of centrifugation. The final cell pellet was resuspended in 5 mL PBS to obtain a single-cell suspension.

The prepared lung single-cell suspension was then subjected to downstream applications such as flow cytometry or single-cell RNA sequencing.

#### Mouse alveolar organoid culture

AT2 cells were purified from single-cell lung suspensions by fluorescence-activated cell sorting (FACS), using GFP expression from the Rosa26-mTmG reporter to identify lineage-labeled AT2 cells. A total of 10,000 DAPI-negative AT2 cells were resuspended in 100 μL of organoid culture medium, mixed thoroughly with 100 μL of growth factor-reduced Matrigel, and seeded as 20 μL droplets into a 24-well plate. After inverting the plate and incubating at room temperature for 20 minutes to allow Matrigel polymerization, 1.5 mL of organoid culture medium containing 10 μM ROCK inhibitor (Y-27632, MCE; HY-U00351) was gently added to each well. Medium was replaced every other day.

The organoid culture medium was prepared using DMEM/F12 as the basal medium and supplemented with the following components: 10 μg/ml Insulin, 5 μg/ml Transferrin, 30 μg/ml Bovine Pituitary Extract(MACGENE; CC203), 50 nM Retinoic Acid(Sigma; R2625), 0.1 mg/mL Cholera Toxin (Sigma; C8052), 50 ng/mL FGF7 ( MCE; HY-P7176), 50 ng/mL Noggin (Novoprotein; C028), 50 ng/mL EGF ( MCE; HY-P7067), 10 μM SB431542 (MCE; HY-10431), and Fetal Bovine Serum (FBS, 5%).

#### Mouse AT2 2D cell culture

FACS-purified GFP^+^ AT2 cells obtained as described above were resuspended in AT2 2D culture medium, and 5 × 10⁴ cells were plated in 100 μL medium onto 48-well plates (or 9-mm coverslips) pre-coated with Geltrex (Thermo Fisher) for 2 hours at 37 °C. During the first 48 hours, cultures were supplemented with 10 μM ROCK inhibitor (Y-27632) to enhance cell survival and attachment. The medium was refreshed every other day.

The AT2 2D culture medium consisted of DMEM/F12 buffered with HEPES, supplemented with 10% FBS and 1× ITS (Insulin-Transferrin-Selenium).

#### Human alveolar organoid culture

Human alveolar organoids were derived from embryonic stem cells (ESCs) as previously described^33^. Upon generation of induced alveolar organoids, further maturation was promoted using Ham's F12 medium supplemented with the following components: 50 nM Dexamethasone (Sigma; D4902), 100 nM 8-Br-cAMP (Sigma-Aldrich; D4902), 100 nM 3-isobutyl-1-methylxanthine (IBMX, Wako; 09503413), 10 ng/mL keratinocyte growth factor (KGF, Novoprotein; CM88;), and 0.1% ITS (Insulin-Transferrin-Selenium supplement, ITS premix; Corning; 354351). Organoids were cultured under 3D conditions in Matrigel and the medium was changed every 4-5 days. Organoids were passaged every 4- 7 days by mechanical dissociation and re-embedding into fresh Matrigel domes.

For pharmacological inhibition experiments, 2,4-PDCA was added to the organoid culture medium at a final concentration of 2 μM and maintained continuously throughout the treatment period. Culture medium was refreshed at regular intervals to ensure stable exposure.

#### Tissue processing and histological staining

Mouse lungs were perfused and fixed in 4% paraformaldehyde (PFA) overnight at 4 °C. After fixation, tissues were processed for either paraffin embedding or cryopreservation, depending on downstream applications. For paraffin embedding, the largest lung lobe was dehydrated using a standard graded ethanol-xylene protocol and embedded in paraffin. Sections (5 μm) were cut, mounted onto glass slides, and baked at 60 °C for 2 hours before staining.

**Hematoxylin and eosin (H&E) staining** was performed using standard protocols. Briefly, paraffin sections were deparaffinized, rehydrated, and stained with hematoxylin and eosin, followed by dehydration and mounting.

**Masson's trichrome staining** was carried out to evaluate pulmonary fibrosis. The staining differentiated nuclei (black), cytoplasm and muscle (red), and collagen fibers (blue), based on the chemical affinities of different dyes to specific tissue components.

Slides were mounted using neutral balsam and imaged for histological analysis.

#### Immunofluorescence staining

Paraffin-embedded or cryosectioned lung tissues were subjected to standard immunofluorescence staining. Briefly, sections were washed in PBS, followed by antigen retrieval (for paraffin sections) using sodium citrate buffer. After blocking and permeabilization with 5% normal donkey serum and 0.1% Triton X-100 in PBS, sections were incubated with primary antibodies overnight at 4°C. The following day, samples were washed and incubated with fluorophore-conjugated secondary antibodies (1:500) for 2 hours at room temperature. Nuclei were counterstained with DAPI, and slides were mounted with antifade mounting medium. Images were acquired using a Nikon A1R-si confocal microscope and processed using Fiji (ImageJ). Quantification and cell counting were performed on maximum intensity projections from Z-stack images.

Detailed information for all primary and secondary antibodies, including sources, catalog numbers, and dilutions, has been compiled in Supplementary Table 2.

#### EdU labeling and detection

Cell proliferation was assessed using EdU (5-ethynyl-2'-deoxyuridine) incorporation. Mice received an intraperitoneal injection of EdU (100 mg/kg) 4 hours before sacrifice. For cultured cells, EdU was added to the medium at a final concentration of 10 μM. After fixation and permeabilization, EdU-positive cells were detected using a Click-iT™ EdU Imaging Kit (Beyotime) according to the manufacturer's instructions. Nuclei were counterstained with DAPI. For tissue sections, EdU detection was performed after immunofluorescence staining. To avoid quenching of fluorescent proteins, EdU labeling and fluorescent protein imaging were conducted in separate channels or experiments when necessary.

#### RNA-seq, m^6^A-seq and data analyses

Total RNA was extracted from FACS-purified AT2 cells isolated on day 14 after bleomycin-induced lung injury. Total RNA (1 μg per sample) was extracted from FACS-purified AT2 cells and submitted to Azenta Life Sciences for bulk RNA sequencing. A total of 10 μg of RNA per sample was submitted to Epibiotek Co., Ltd. for m^6^A RNA immunoprecipitation sequencing (MeRIP-seq).

For RNA-Seq, the raw paired-end reads were preprocessed to remove the adapters with TrimGalore version 0.6.10. Processed reads were mapped against the ribosomal RNA sequence download from NCBI and used the unmapped sequences for further analysis in order to remove ribosomal RNA sequences. The sequences were mapped to mm10.p6 mouse genome using HISAT2 aligner and removed duplicates with sambamba. The exomePeak2 R package were used for peak calling and differential peak analysis.

For MeRIP-seq, the raw paired-end reads were preprocessed to remove the adapters with TrimGalore version 0.6.10. Processed reads were mapped against the mouse genome (mm10.p6) using HISAT2 aligner (v2.2.1)^34^. The uniquely mapped reads that passed the "markdup-r" filter by sambamba (v1.0.0)^35^ were kept to quantify gene expression. The generation of gene counts (reads aligned to each gene of each sample) was performed using mm10.p6_NCBI_108.gtf and FeatureCounts (v2.0.3)^36^. Differential gene expression was analyzed using the DESeq2 version 1.30.1 R package. DEGs were identified by setting a cutoff of Padj value < 0.05 and fold change > ±2.

#### scRNA-seq and data analyses

For single-cell RNA sequencing, 40% FACS-purified GFP^+^ AT2 cells were mixed with 60% whole-lung cells. After confirming cell viability above 85% using trypan blue staining, the suspension was processed using the Chromium Single Cell 3' v3.1 platform (10x Genomics) at Novogene. Sequencing data were aligned and quantified using the Cell Ranger single-cell software suite (version 6.1.2, 10x Genomics) against the provided mm10 mouse reference genome. Ambient RNA were removed by CellBender^37^.

scRNA-seq data were analysed in Python using the standard scanpy workflows. Quality control for doublets were using scrublet with default parameters. High-quality cells were obtained by excluding cells with less than 25000 transcripts or 6000 genes and those with a higher than 15% mitochondrial gene content. Then mitochondrial and ribosomal genes were excluded from analysis. Following log-normalization of count data, the top 2,000 highly variable genes were selected per sample with the "cell_ranger" approach. Principal components derived from these genes were then integrated via harmony. For clustering analysis, a shared nearest neighbor graph was constructed and the modularity function was optimized using the Leiden algorithm. Cells were first classified into broad categories—epithelial, endothelial, mesenchymal, and immune—and were then further subclustered within each major type. Pathway activity scores were calculated using the decoupler (Python version) package with PROGENy^38^ gene signatures. Diffusion pseudotime was computed using default parameters. The root cells were defined as those with the minimum values in the first diffusion component to establish the start of the trajectory.

#### qPCR and MeRIP-qPCR

To validate m^6^A-modified target transcripts regulated by Alkbh5, we performed m^6^A RNA immunoprecipitation followed by quantitative PCR (MeRIP-qPCR).

Briefly, total RNA was extracted using TRIzol (Invitrogen), quantified by Qubit, and fragmented at 94°C for 5 minutes. Fragmented RNA was incubated with anti-m^6^A antibody (5 μg per 100 μg RNA) overnight at 4°C, followed by pulldown using protein A/G magnetic beads. After washing, the m^6^A-enriched RNA was eluted by Proteinase K digestion and purified using an RNA Clean & Concentrator kit (Zymo Research).

Purified RNA was reverse transcribed using random hexamer primers. Quantitative PCR (qPCR) was performed with SYBR Green Master Mix and gene-specific primers. Enrichment of m^6^A-modified transcripts was calculated using the ΔΔCt method by comparing MeRIP samples to input controls.

#### mRNA stabilization assay

AT2-derived alveolar organoids from control and *Alkbh5* knockout mice were cultured for 14 days. Transcription was halted by adding actinomycin D (5 μg/mL) to the culture medium. Organoids were collected at 0, 2-, 4-, 6-, and 8-hours post-treatment. Total RNA was extracted using TRIzol (Invitrogen), quantified by NanoDrop, and reverse-transcribed into cDNA. Quantitative PCR was performed to measure Areg mRNA levels at each time point. mRNA half-life was calculated by fitting the decay curve to an exponential model.

#### Statistical analysis

All experiments were performed with at least three independent biological replicates unless otherwise indicated, and data are presented as mean ± standard deviation (SD). Sample sizes were determined based on prior experience and published studies in similar experimental systems, ensuring sufficient statistical power to detect biologically meaningful differences. Statistical analyses were conducted using GraphPad Prism 8. Prior to parametric testing, data distribution was assessed for normality using the Shapiro-Wilk test, and homogeneity of variances was evaluated using F-test. For comparisons between two groups, unpaired two-tailed Student's t-test was used. For experiments involving more than two groups, one-way analysis of variance (ANOVA) followed by appropriate post hoc tests (e.g., Tukey's test) was applied when necessary. A P value of less than 0.05 was considered statistically significant. Detailed statistical methods are specified in the corresponding figure legends.

## Results

### Changes in Alkbh5 expression across lung cell types under homeostatic and injury conditions

To define the cellular context of Alkbh5 expression in the lung, we first analyzed published single-cell RNA-seq dataset^39^. Alkbh5 was detected across multiple lung cell populations under both homeostatic and bleomycin (BLM)-induced injury conditions, with changes observed following injury (Fig. S1A). To validate these findings, we performed immunofluorescence co-staining in lung sections. Alkbh5 expression was examined in major cell populations, including AT2 cells (pro-SPC^+^), AT1 cells (PDPN^+^), club cells (CC10^+^), mesenchymal cells (α-SMA^+^), and macrophages (F4/80^+^) (Fig. S1B, C). Alkbh5 was broadly expressed across these cell types and exhibited cell type-dependent changes following injury, with significant alterations observed in most populations except club cells. We observed a significant decrease in Alkbh5 expression in AT2 cells following injury, as demonstrated by both single-cell RNA-seq analysis and immunofluorescence staining.

Given its prominent expression and regulation in AT2 cells, we next investigated the functional role of Alkbh5 in AT2-mediated alveolar homeostasis and regeneration.

### Alkbh5 is dispensable for AT2 cell homeostasis

We generated AT2-specific* Alkbh5* conditional knockout mice (*Alkbh5^fl/fl^; Sftpc-CreERT2; Rosa26-mTmG)* by genetic crossing in order to investigate the role of Alkbh5 in AT2 cells. Upon tamoxifen induction,* Alkbh5* was specifically deleted in AT2 cells, which simultaneously expressed membrane-bound EGFP. The labeling efficiency was approximately 95%, as confirmed by fluorescence imaging (Fig. S2A, B). GFP^+^ AT2 cells were subsequently isolated by flow cytometry, and qRT-PCR analysis verified robust loss of Alkbh5 mRNA. Immunofluorescence staining further confirmed the absence of ALKBH5 protein in knockout lungs (Fig. S2D-F).

To assess the role of* Alkbh5* in maintaining AT2 cell function under homeostatic conditions, we used *Alkbh5^fl/fl^; Sftpc-CreERT2; Rosa26-Brainbow* mice and corresponding heterozygous controls (*Alkbh5^fl/+^*) treated with tamoxifen. One year after induction, lungs were collected for phenotypic analysis. Histological examination revealed no overt morphological changes in Alkbh5-deficient lungs compared to controls (Fig. S3A). Quantification of mean alveolar size showed no significant differences (Fig. S3B). Immunofluorescence staining for Ki67 demonstrated comparable levels of AT2 cell proliferation between groups (Fig. S3C). Furthermore, clonal analysis using the Brainbow reporter showed no significant alterations in clone size or expansion (Fig. S3D). We also assessed the presence of transitional (Krt8^+^) and differentiated AT1 (PDPN^+^) cells, and again observed no significant differences (Fig. S3E). Together, these results indicate that Alkbh5 deletion in AT2 cells does not disrupt alveolar epithelial homeostasis in the steady-state lung.

### Alkbh5 deletion enhances AT2-mediated alveolar repair in diverse lung injury models

**Figure 1:**
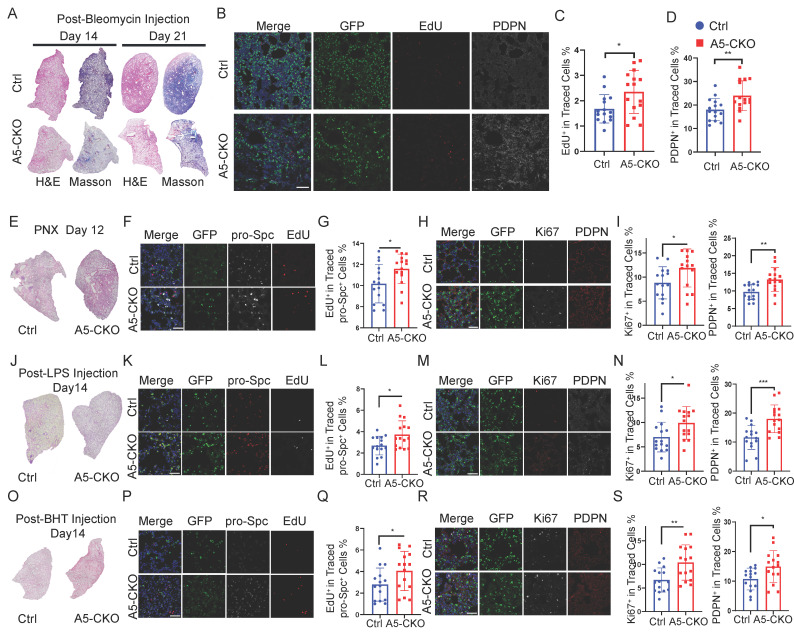
** Alkbh5 deletion promotes AT2-driven repair in diverse lung injury models.** (A-D) Bleomycin-induced lung injury model. (A) H&E and Masson's trichrome staining of the left lung lobe on day 14 and day 21 post-bleomycin injury. (B) Immunofluorescence staining of lineage-traced AT2 cells (GFP, green) for proliferating cells (EdU, red) and differentiated AT1 cells (PDPN, white) at day 21 with scale bars representing 100 μm. (C, D) Quantification of proliferating (EdU^+^) and differentiated (PDPN^+^) AT2 cells from (B). (E-I) PNX model. (E) H&E staining of lungs at day 12 post-PNX. (F) Immunofluorescence staining of lineage-labeled AT2 cells (GFP, green), AT2 marker (pro-Spc, white) and proliferating cells (EdU, red) with scale bars representing 50 μm. (G) Quantification of EdU^+^ AT2 cells. (H) Immunostaining for Ki67 (white) and PDPN (red) in lineage-labeled AT2 cells (GFP, green) with scale bars representing 50 μm. (I) Quantification of Ki67^+^ and PDPN^+^ cells from (H). n = 5 mice (J-N) LPS-induced acute lung injury model. (J) H&E staining of lungs at day 14 post-LPS injury. (K) Immunofluorescence staining of lineage-traced AT2 cells (GFP, green) and proliferating cells (EdU, white) with scale bars representing 50 μm. (L) Quantification of EdU^+^ AT2 cells. (M) Immunostaining for PDPN (red) in lineage-labeled AT2 cells (GFP, green) with scale bars representing 50 μm. (N) Quantification of PDPN^+^ cells from (M). n = 5 mice (O-S) BHT-induced acute lung injury model. (O) H&E staining of lungs at day 14 post-BHT injury. (P) Immunofluorescence staining of lineage-traced AT2 cells (GFP, green) and proliferating cells (EdU, red) with scale bars representing 50 μm. (Q) Quantification of EdU^+^ AT2 cells. (R) Immunostaining for PDPN (red) in lineage-labeled AT2 cells (GFP, green) with scale bars representing 50 μm. (S) Quantification of PDPN^+^ cells from (R). Data are presented as mean ± SD from n = 5 mice per group,* each dot represents an individual field*. Statistical significance was determined using unpaired two-tailed Student's t-test. *P < 0.05, **P < 0.01, ***P < 0.001.

Next, we employed *Rosa26-mTmG* reporter mice, which enable simultaneous AT2-specific *Alkbh5* deletion and lineage labeling following tamoxifen induction, to evaluate the role of Alkbh5 under injury conditions. Mice were subjected to four distinct models of lung injury—bleomycin (BLM), pneumonectomy (PNX), lipopolysaccharide (LPS), and butylated hydroxytoluene (BHT). In the BLM-induced injury model, which induces both epithelial damage and fibrotic remodeling, H&E and Masson's trichrome staining revealed that Alkbh5-deficient lungs exhibited improved tissue repair and attenuated fibrosis compared to controls (Fig. 1A). To further evaluate the effects of* Alkbh5* deletion on AT2 cell behavior, we performed EdU incorporation assays to assess AT2 cell proliferation, and conducted immunostaining for the AT1 cell marker PDPN on lineage-traced cells. The results revealed that Alkbh5-deficient AT2 cells exhibited enhanced proliferation and accelerated differentiation into AT1 cells, indicating a more active contribution to alveolar regeneration (Fig. 1B-D). We next asked whether these pro-regenerative effects of* Alkbh5* loss were conserved across other injury settings. In the PNX model, which induces compensatory alveolar growth through mechanical stretch of the remaining lobes^40^, ^41^, overall lung morphology appeared comparable between genotypes. However, EdU and PDPN staining showed that Alkbh5-deficient AT2 cells exhibited increased proliferation and AT1 differentiation, mirroring findings in the BLM model (Fig. 1E-I).Similarly, in the LPS-induced acute lung injury model, although both groups showed gross morphological recovery by day 7 post-injury, AT2 cells in *Alkbh5*-deficient mice again demonstrated increased proliferative capacity and enhanced differentiation into AT1 cells (Fig. 1J-N), suggesting a robust regenerative phenotype in an inflammation-driven context. In the BHT-induced injury model, which mimics oxidative epithelial damage, we observed that Alkbh5-deficient AT2 cells exhibited enhanced regenerative capacity, accompanied by increased EdU incorporation and AT1 marker expression, further supporting enhanced proliferative and differentiative capacity (Fig. 1O-S). Collectively, these results demonstrate that Alkbh5 deletion consistently enhances AT2-mediated alveolar regeneration across diverse models of lung injury, underscoring its conserved role as a negative regulator of epithelial repair.

### *Alkbh5* knockout enhances AT2 cell proliferation and differentiation *in vitro*

**Figure 2:**
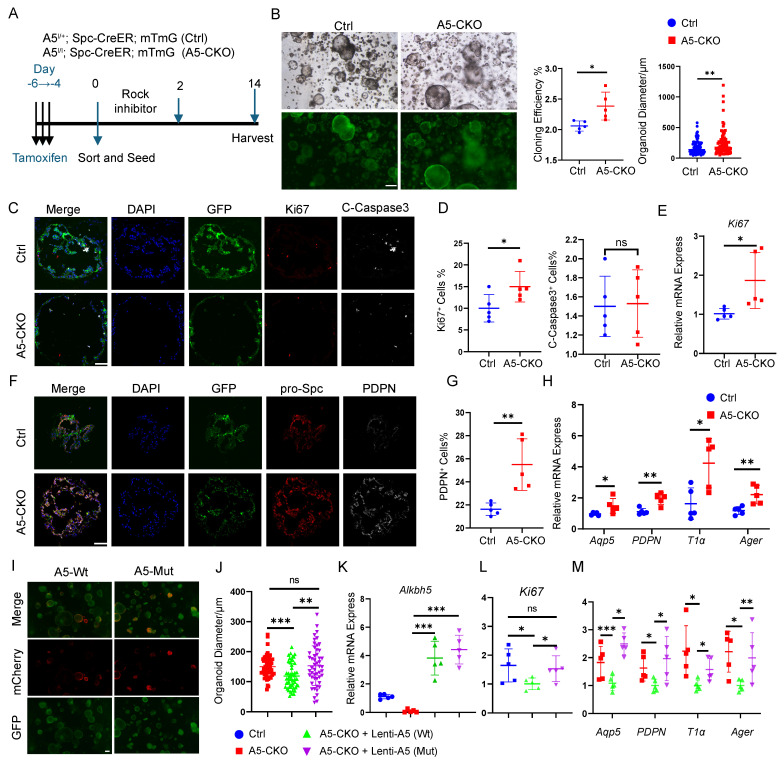
** Alkbh5 deletion in AT2 cells enhances proliferation and differentiation *in vitro*.** (A) Schematic diagram of the alveolar organoid assay. FACS-purified GFP^+^ AT2 cells from control and Alkbh5-deficient mice were embedded in Matrigel and cultured for 14 days. (B) Bright-field and GFP fluorescence images of day-14 organoids derived from control and Alkbh5-deficient AT2 cells. Scale bars, 100 μm. Quantification of organoid forming efficiency and organoid diameter demonstrates. Clonogenic efficiency was calculated across five independent experiments (n = 5). Organoid diameter was quantified from a total of 90 organoids, with each data point representing an individual organoid. Statistical significance was determined using unpaired two-tailed Student's t-test. Data are presented as mean ± SD. *P < 0.05, **P < 0.01. (C) Immunofluorescence staining of frozen organoid sections showing GFP (lineage label), Ki67 (proliferation marker), and Cleaved Caspase-3 (apoptosis marker). Scale bars, 100 μm. (D) Quantification of Ki67^+^ proliferating cells and Cleaved Caspase-3^+^ apoptotic cells shown in (C). Data are presented as mean ± SD, * p<0.05; ns, not significant (unpaired, two-tailed Student's t-test). (E) qRT-PCR analysis of *Ki67* expression in control and Alkbh5-deficient organoids. Data are presented as mean ± SD. * p<0.05 (unpaired, two-tailed Student's t-test). (F) Immunofluorescence staining of organoid sections illustrating GFP-labeled lineage-traced cells, pro-SPC (AT2 marker), and PDPN (AT1 marker). Scale bars, 100 μm. (G) Quantification of the proportion of pro-SPC^+^ AT2 cells and PDPN^+^ AT1 cells shown in (F). Data are presented as mean ± SD, **P < 0.01 (unpaired, two-tailed Student's t-test). (H) qRT-PCR analysis of AT1 lineage markers in control versus Alkbh5-deficient organoids. Data are presented as mean ± SD, *P < 0.05, **P < 0.01 (unpaired, two-tailed Student's t-test). (I) Fluorescence images of Alkbh5-deficient AT2-derived organoids transduced with lentiviruses expressing mCherry-tagged Alkbh5-WT or catalytically inactive Alkbh5-Mut. Scale bars, 100 μm. (J) Quantification of organoid size after rescue with Alkbh5-WT or Alkbh5-Mut. Data are presented as mean ± SD, **P < 0.01, ***P < 0.001; ns, not significant (unpaired, two-tailed Student's t-test). (K) qRT-PCR validation of Alkbh5 expression levels in sorted mCherry^+^ cells from rescue organoids. Data are presented as mean ± SD, ***P < 0.001 (unpaired, two-tailed Student's t-test). (L) qRT-PCR analysis of Ki67 expression sorted mCherry^+^ cells. Data are presented as mean ± SD, * p<0.05; ns, not significant (unpaired, two-tailed Student's t-test). (M) qRT-PCR analysis of AT1 marker gene expression in sorted mCherry^+^ cells. Data are presented as mean ± SD, * p<0.05 (unpaired, two-tailed Student's t-test).

We conducted alveolar organoid assays using FACS-purified AT2 cells to investigate the intrinsic regenerative potential of AT2 cells *in vivo*. Organoids were cultured for 14 days, during which spherical alveolar-like structures developed while maintaining lineage-tracing fluorescence (Fig. 2A, B). Quantitative analysis revealed a significant increase in both the number and size of organoids derived from Alkbh5-deficient AT2 cells, compared to wild-type controls (Fig. 2B). We next assessed proliferative and apoptotic activity within the organoids. Immunofluorescence staining for Ki67 and cleaved caspase-3 showed that Alkbh5 deletion markedly increased AT2 proliferation without affecting apoptosis (Fig. 2C-D). Consistently, qRT-PCR analysis confirmed elevated Ki67 expression in Alkbh5-deficient organoids (Fig. 2E). To evaluate epithelial differentiation, we examined AT1 and AT2 markers within organoids. Alkbh5-deficient organoids contained a higher proportion of PDPN^+^ AT1-like cells (Fig. 2F-G), and RNA analysis further demonstrated increased expression of multiple AT1-associated genes (Fig. 2H). These results indicate that loss of Alkbh5 not only enhances AT2 proliferation but also accelerates their differentiation toward the AT1 lineage.

To determine whether these phenotypes depend on the m^6^A demethylase activity of Alkbh5, we generated lentiviral constructs expressing either wild-type Alkbh5 (Alkbh5-WT) or a catalytically inactive mutant (H205A, Alkbh5-Mut)^42^, and reintroduced them into Alkbh5-deficient AT2 cells prior to organoid culture. Successful transduction was confirmed by mCherry fluorescence (Fig. 2I). Organoids expressing Alkbh5-WT were markedly smaller than those expressing Alkbh5-Mut (Fig. 2J). Sorted mCherry^+^ cells exhibited clear re-expression of Alkbh5 at the RNA level (Fig. 2K). Restoration of Alkbh5-WT—but not the catalytic mutant—suppressed proliferation (Fig. 2L) and reduced AT1 marker expression (Fig. 2M), thereby reversing the phenotypes observed in Alkbh5-deficient AT2 cells.

Together, these data demonstrate that Alkbh5-mediated suppression of AT2 proliferation and differentiation is dependent on its enzymatic activity in m^6^A demethylation, underscoring the functional relevance of m^6^A dynamics in alveolar epithelial regeneration.

### Alkbh5 suppresses AT2 activation by restricting Areg secretion from transitional cells

**Figure 3:**
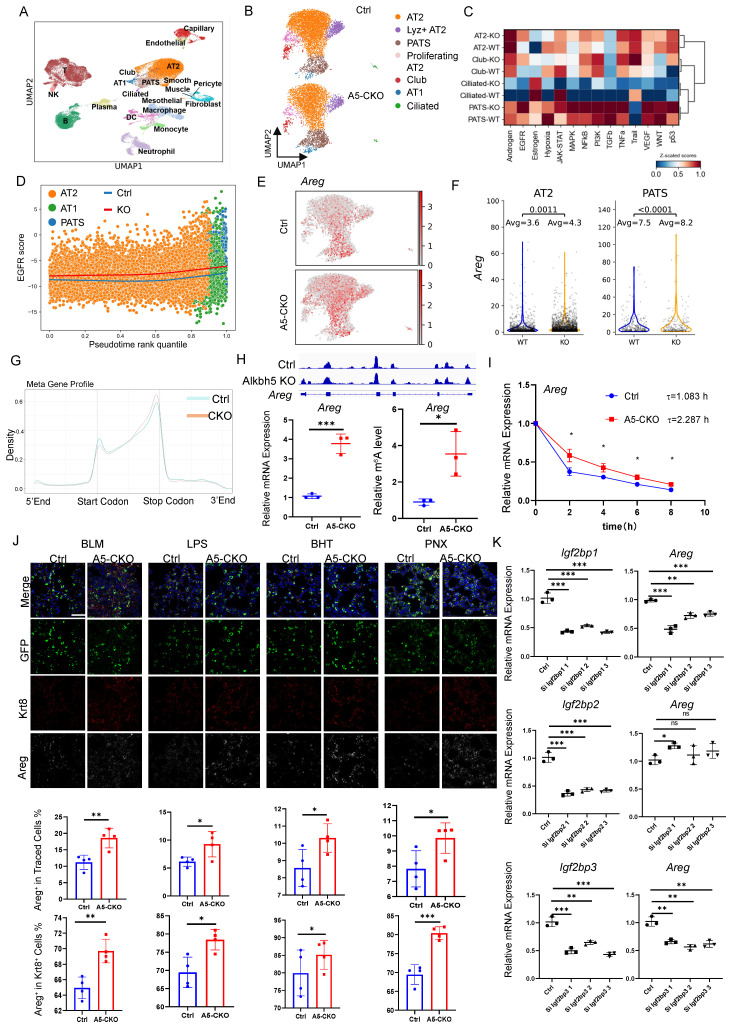
** Alkbh5 limits AT2 proliferation and differentiation by suppressing Areg secretion from PATs.** (A) UMAP plot showing major lung cell populations identified by scRNA-seq at day 14 after bleomycin injury. Single-cell suspensions were prepared from lungs of four mice (two control and two AT2-specific Alkbh5 knockout mice) by combining 40% EGFP^+^ lineage-labeled epithelial cells with 60% total lung cells prior to sequencing. (B) Refined UMAP clustering of EGFP^+^ epithelial cells from Ctrl and A5-CKO lungs. Cells are grouped into distinct epithelial populations, including AT2, transitional AT2 (PATS), proliferating AT2, AT1, club, and ciliated cells. (C) Pathway enrichment analyses of major epithelial clusters—including AT2, PATS, Club, and Ciliated cells—highlighting injury-induced changes in key signaling pathways. (D) EGFR pathway activity scores across AT2, PATS, and AT1 populations, demonstrating enhanced EGFR signaling activation following Alkbh5 deletion. (E) Feature plot showing Areg expression across EGFP^+^ epithelial cells in Ctrl and Alkbh5-CKO groups. (F) Violin plots illustrating Areg expression levels specifically within AT2 and PATS subsets under control and Alkbh5-deficient conditions. Statistical significance was determined using unpaired two-tailed Student's t-test. (G) MeRIP-seq metagene profiles showing global m⁶A peak distribution along transcripts in AT2 cells, comparing control and Alkbh5-deficient groups. (H) Genome browser view of m^6^A peaks on the Areg gene locus. Quantification of Areg mRNA expression by RT-qPCR and m^6^A methylation levels by MeRIP-qPCR in control and Alkbh5-deficient AT2 cells. Data are presented as mean ± SD, *P < 0.05, ***P < 0.001 (unpaired, two-tailed Student's t-test). (I) mRNA stability assay for Areg after actinomycin D treatment in control and Alkbh5-deficient AT2-derived organoids. Statistical significance at each time point was determined using an unpaired two-tailed Student's t-test. *P < 0.05.(J) Representative immunofluorescence staining showing Areg (white), Krt8 (marker of transitional AT2 cells, red), and GFP (lineage marker, green) across different lung injury models (BLM, PNX, LPS, and BHT). Scale bars, 50 μm. Data are presented as mean ± SD from n = 5 mice per group. Statistical significance was determined using unpaired two-tailed Student's t-test. *P < 0.05, **P < 0.01, ***P < 0.001. (K) *Areg* expression levels in MLE12 cells after siRNA-mediated knockdown of I*gf2bp1, Igf2bp2,* and* Igf2bp3.* Data are presented as mean ± SD, * *p*<0.05, *** *p* <0.001, **** *p*<0.0001 (one-way ANOVA).

To examine transcriptional changes after Alkbh5 deletion, we performed scRNA-seq on day 14 after bleomycin injury. To ensure adequate resolution of rare AT2 subpopulations, we mixed 40% lineage-labeled cells with 60% unsorted whole-lung cells prior to sequencing. Clustering analysis revealed no emergence of novel cell types, and the overall cellular composition remained comparable between control and knockout lungs (Fig. 3A). While the overall cellular composition was largely comparable between control and knockout lungs, the knockout group showed an increase in PATS cell abundance (Fig. 3B). We next performed higher-resolution sub-clustering of EGFP^+^ lineage-labeled cells (Fig. 3B; Fig. S4A-B) and conducted differential gene expression analysis in AT2 and pre-alveolar type 1 transitional state (PATS) cells (Fig. S4C). Pathway enrichment analysis showed pronounced alterations in EGFR signaling, particularly within AT2 and PATS populations in Alkbh5-deficient lungs (Fig. 3C). Along the inferred AT2→PATS→AT1 differentiation trajectory, Alkbh5 deletion was associated with progressive and robust activation of EGFR signaling (Fig. 3D; Fig. S4D). We then analyzed EGFR ligands and found that *Areg* expression was markedly increased in both AT2 and PATS cells following *Alkbh5* deletion (Fig. 3E-F). *EGFR* expression was detected in both populations (Fig. S4E-F), suggesting a potential autocrine/paracrine regulatory loop. In terms of expression distribution, Areg was more widespread in the Alkbh5-deficient group compared to controls, whereas EGFR expression remained largely unchanged. To further define the molecular mechanism, we performed RNA-seq and MeRIP-seq on FACS-isolated AT2 cells collected 14 days after bleomycin injury. As expected, the global m⁶A distribution pattern conformed to canonical features (Fig. 3G), with locally elevated m⁶A peaks in the Alkbh5-deficient group. Integrated MeRIP-seq and RNA-seq analyses identified Areg as a common target, exhibiting increased m⁶A methylation and elevated transcript abundance (Fig. S4G). These findings were confirmed by MeRIP-qPCR (Fig. 3H). In addition to Areg, multiple regeneration-associated genes exhibited coordinated changes in m⁶A modification and transcript abundance following Alkbh5 deletion (Fig. S4H). Among these candidates, Areg was prioritized for further mechanistic investigation.

To test whether m⁶A methylation contributes to Areg transcript stability, we performed actinomycin D chase experiments in primary AT2-derived organoids. Consistent with increased methylation, Alkbh5-deficient organoids exhibited a prolonged half-life of *Areg* mRNA (τ = 2.287) compared with controls (τ = 1.083) (Fig. 3I). Validation across all four injury models—BLM, PNX, LPS, and BHT—consistently demonstrated elevated Areg expression in AT2 cells, with the strongest induction observed in transitional AT2 subsets (Fig. 3J).

Finally, we sought to identify potential m⁶A reader proteins responsible for regulating Areg mRNA stability. We next performed siRNA-mediated knockdown of Igf2bp1, Igf2bp2, and Igf2bp3 in the AT2 cell line MLE12. Notably, silencing of Igf2bp1 and Igf2bp3, but not Igf2bp2, led to a marked reduction in Areg expression (Fig. 3K), suggesting that these IGF2BP family members may function as m⁶A readers that stabilize Areg transcripts.

Collectively, these results demonstrate that Alkbh5 restricts AT2 activation by suppressing Areg expression through m⁶A-dependent regulation of mRNA stability, with IGF2BP1 and IGF2BP3 emerging as key reader proteins mediating this effect.

### Areg drives AT2 proliferation and differentiation via autocrine signaling after injury

**Figure 4:**
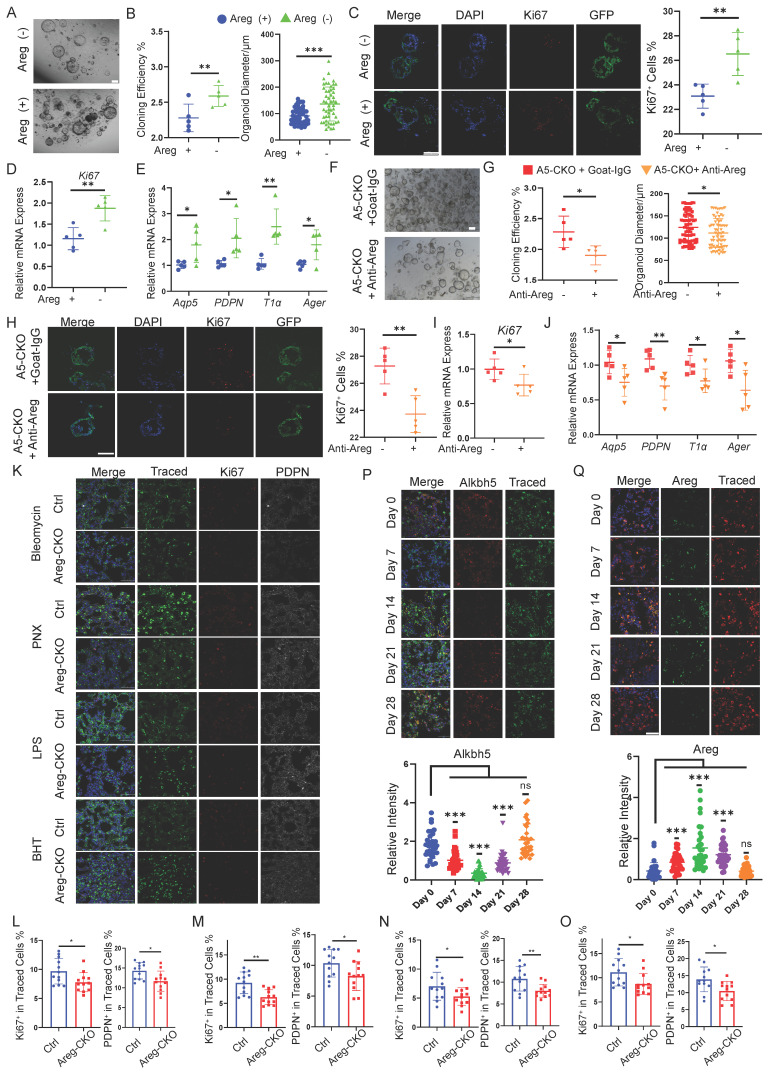
** Areg drives AT2 proliferation and differentiation via autocrine signaling.** (A-E) Recombinant Areg stimulation of wild-type AT2-derived alveolar organoids. (A) Bright-field images of wild-type AT2-derived organoids cultured with or without recombinant Areg for 14 days. Scale bar, 100 μm.(B) Quantification of organoid formation efficiency and organoid diameter in control versus Areg-treated cultures. (C) Immunofluorescence staining of organoid frozen sections showing Ki67^+^ proliferating cells, together with the corresponding quantification. Scale bar, 100 μm. (D) RT-qPCR analysis of proliferation-associated gene Ki67 in control and Areg-treated organoids. (E) RT-qPCR analysis of AT1 lineage marker expression in organoids cultured with or without recombinant Areg. Clonogenic efficiency was calculated across five independent experiments (n = 5). Organoid diameter was quantified from a total of 50-80 organoids, with each data point representing an individual organoid. Statistical significance was determined using unpaired two-tailed Student's t-test. Data are presented as mean ± SD. *P < 0.05, **P < 0.01, ***P < 0.001. (F-J) Neutralization of Areg in Alkbh5-deficient AT2 organoid cultures. (F) Bright-field images of alveolar organoids derived from Alkbh5-deficient AT2 cells cultured for 14 days in the presence or absence of an Areg-neutralizing antibody. Scale bar, 100 μm. (G) Quantification of organoid formation efficiency and organoid diameter in cultures treated with neutralizing antibody or control IgG. (H) Immunofluorescence staining of frozen organoid sections showing Ki67^+^ proliferating cells and quantification of Ki67^+^ cell proportions. Scale bar, 100 μm.(I) RT-qPCR analysis of* Ki67* expression in Alkbh5-deficient organoids treated with neutralizing antibody or control IgG. (J) RT-qPCR analysis of AT1 lineage marker expression in Alkbh5-deficient organoids cultured with neutralizing antibody or control IgG. Clonogenic efficiency was calculated across five independent experiments (n = 5). Organoid diameter was quantified from a total of 50-80 organoids, with each data point representing an individual organoid. Statistical significance was determined using unpaired two-tailed Student's t-test. Data are presented as mean ± SD. *P < 0.05, **P < 0.01. (K) Representative immunofluorescence images showing lineage-traced epithelial cells in control mice (Sftpc-CreER; Rosa26-mTmG) and AT2-specific Areg conditional knockout mice (Areg*^fl/fl^*; Sftpc-CreER; Rosa26-mTmG) after bleomycin, PNX, LPS, and BHT injury. Traced labels lineage-traced AT2-derived cells, Ki67 marks proliferating cells, and PDPN marks AT1 cells. Nuclei were counterstained with DAPI. Scale bars, 50 μm. (L-O) Quantification of Ki67^+^ and PDPN^+^ cells among lineage-traced GFP^+^ cells after bleomycin (L), PNX (M), LPS (N), and BHT (O) injury. Data are presented as mean ± SD. n = 4 mice per group, with four randomly selected fields analyzed per mouse. Each dot represents an individual field. Statistical significance was determined by unpaired two-tailed Student's t-test. *p < 0.05, **p < 0.01. (P, Q) Representative immunofluorescence images showing Alkbh5 (P) and Areg (Q) expression in lineage-traced epithelial cells at different time points (days 0, 7, 14, 21, and 28) following injury. Traced marks lineage-traced epithelial cells. Nuclei were stained with DAPI. Scale bars, 50 μm. Together with the quantification of Alkbh5 and Areg fluorescence intensity at the indicated time points. Data are presented as mean ± SD. n = 4 mice per group, each dot represents an individual field. Statistical significance was determined by one-way ANOVA with appropriate post hoc tests. ns, not significant; ***P < 0.001.

Then we supplemented recombinant Areg into the AT2-derived organoid culture system to further assess the functional role of Areg in AT2 cell behavior. Areg treatment significantly enhanced both organoid size and clonogenic capacity, reaching levels comparable to those observed in Alkbh5-deficient cells (Fig. 4A-B). Immunofluorescence staining and qRT-PCR analysis of Ki67 confirmed that Areg robustly enhanced AT2 organoid proliferation (Fig. 4C-D). In addition, Areg supplementation elevated the expression of AT1 lineage markers, indicating accelerated differentiation (Fig. 4E). Conversely, blockade of Areg signaling with a neutralizing antibody largely suppressed the Alkbh5-deficient phenotype. In Alkbh5-KO AT2 organoids, Areg neutralization significantly reduced organoid growth, diminished proliferative activity, and attenuated AT1 lineage differentiation (Fig. 4F-J), demonstrating that Areg is required for the enhanced regenerative capacity induced by Alkbh5 loss. To further validate the role of Areg in vivo, we generated AT2 cell-specific Areg conditional knockout mice and examined epithelial repair across multiple injury models. Loss of Areg in AT2 cells significantly reduced the proliferation of lineage-traced epithelial cells and impaired their differentiation into AT1 cells following bleomycin, PNX, LPS, and BHT-induced injury (Fig. 4K-O). Together, these findings establish that Areg functions as a key autocrine mediator downstream of Alkbh5 to promote AT2 activation and alveolar regeneration.

We next examined the temporal dynamics of Alkbh5 and Areg expression during lung repair. Immunofluorescence analysis revealed that Areg expression was elevated at early to intermediate stages following injury, whereas Alkbh5 expression showed an opposite trend, gradually recovering at later stages (Fig. 4P, Q). This inverse pattern suggests a temporally coordinated regulation of the Alkbh5-Areg axis during alveolar regeneration.

Together, these results demonstrate that Areg functions as a key autocrine mediator downstream of Alkbh5 to promote AT2 activation, with its activity dynamically regulated during lung repair.

### Areg enhances proliferation and differentiation of AT2 and activates EGFR

**Figure 5:**
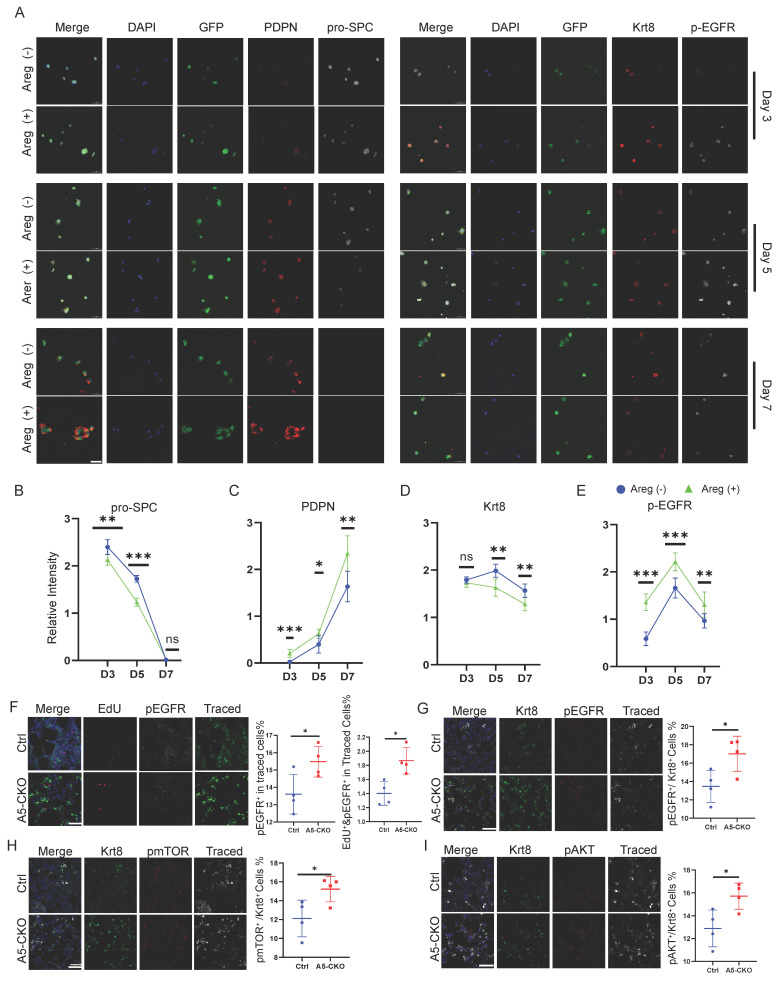
** Areg activates EGFR and accelerates AT2 proliferation and differentiation.** (A) Immunofluorescence staining of 2D-cultured AT2 cells treated with recombinant AREG and collected at days 3, 5, and 7. GFP marks lineage-labeled AT2-derived cells. Left panels show staining for the AT1 marker PDPN and the AT2 marker pro-SPC. Right panels show staining for the transitional AT2 (PATS) marker Krt8 and phosphorylated EGFR (p-EGFR), indicating EGFR pathway activation. Scale bars, 50 μm. (B-E) Quantification of marker expression corresponding to (A) (B) pro-SPC (AT2 marker), (C) PDPN (AT1 marker), (D) Krt8 (transitional AT2/PATS marker), (E) p-EGFR (EGFR pathway activation). All values are presented as mean ± SD; *p < 0.05, **p < 0.01, ***p < 0.001; nonsignificant (NS) (unpaired, two-tailed Student's t-test). (F) Immunofluorescence staining of lung sections on day 14 after bleomycin injury. EdU (red) labels proliferating cells, p-EGFR (white) marks EGFR activation, and Traced (green) indicates lineage-labeled AT2 cells. (G-I) Immunofluorescence staining and quantification of p-EGFR (red), p-mTOR (red), and p-AKT (red) activation in transitional AT2 cells (Krt8^+^, green) following bleomycin injury. Traced (white) marks lineage-labeled cells. Scale bars, 50 μm. Data are presented as mean ± SD from n =4 mice per group; analyzed by Two-tailed unpaired Student's t test, *P < 0.05.

We next examined this process in a more temporally resolved manner using primary AT2 cells cultured under 2D conditions (Fig. 5A). Quantification of AT2, AT1, and transitional PATS markers, together with analysis of EGFR pathway activation (Fig. 5B-D), revealed that Areg accelerates the progression of transitional AT2 cells toward the AT1 fate, accompanied by increased EGFR signaling activity (Fig. 5E). We then evaluated EGFR pathway activation *in vivo*. In the bleomycin injury model, phosphorylation of EGFR was markedly increased in lineage-labeled AT2 cells from Alkbh5-deficient lungs, and EGFR-activated cells exhibited higher proliferative activity (Fig. 5F). Downstream pathway analysis further confirmed robust activation of EGFR signaling in the knockout group (Fig. 5G-I). Notably, a greater proportion of Krt8^+^ transitional AT2 cells displayed EGFR pathway activation in the absence of Alkbh5. Consistent with this, similar patterns of EGFR activation were observed across the PNX, LPS, and BHT injury models (Fig. S5), supporting a conserved role for the Areg-EGFR axis in regulating AT2 activation and repair across diverse injury contexts.

These findings suggest that Alkbh5 suppresses AT2 activation and alveolar regeneration at least in part by restricting Areg-mediated EGFR signaling in transitional epithelial cells.

### Deletion of ALKBH5 enhances proliferation and differentiation of human alveolar organoids

**Figure 6:**
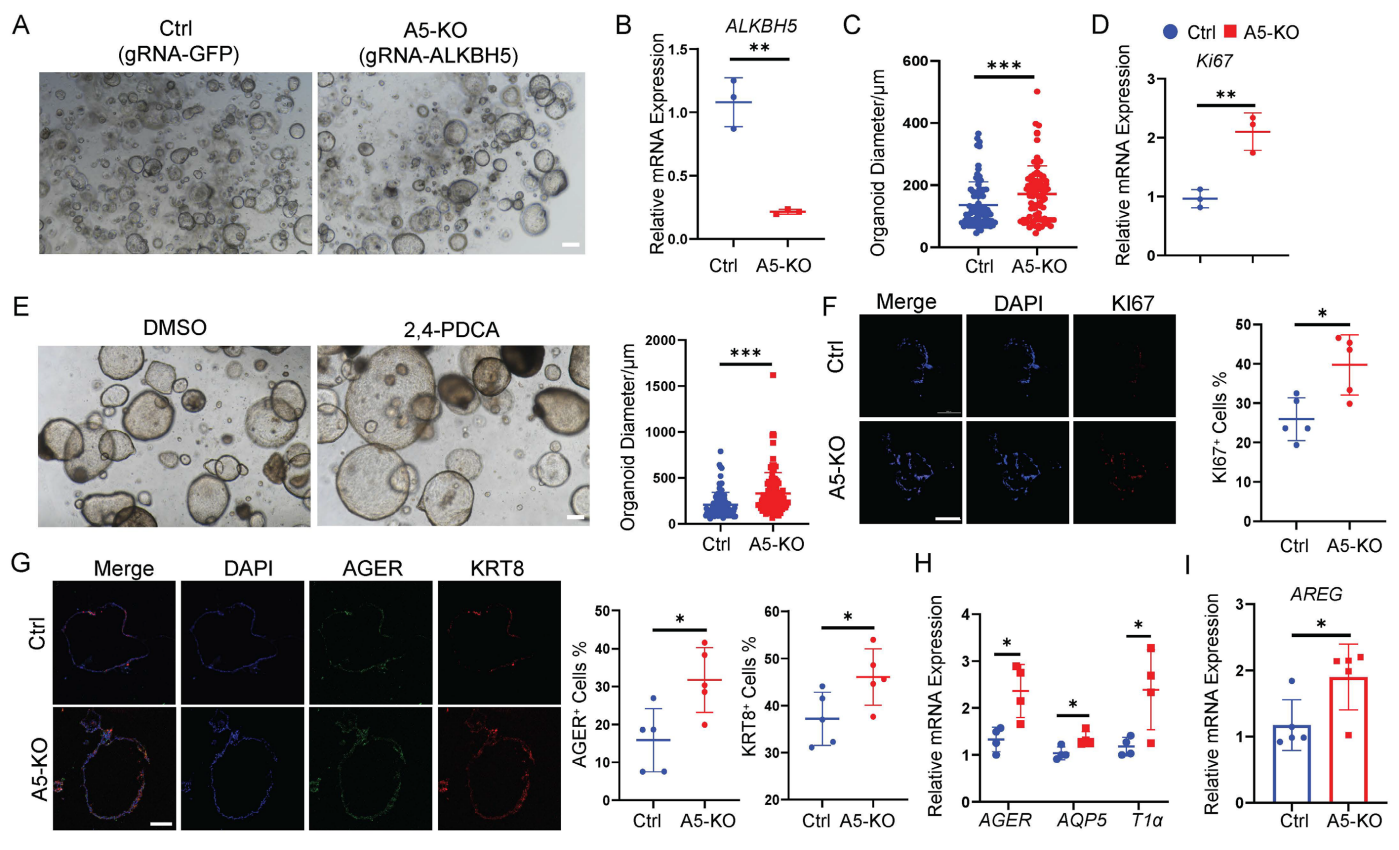
** Deletion of ALKBH5 enhances proliferation and differentiation of human alveolar organoids.** (A) Bright-field images of human alveolar organoids derived from control (gRNA-GFP) and ALKBH5-knockout (gRNA-ALKBH5) ESC-derived cultures. Scale bar, 100 μm. (B) RT-qPCR analysis of ALKBH5 mRNA expression in control and ALKBH5-knockout organoids. Organoid diameter was quantified from a total of 80 organoids, with each data point representing an individual organoid. Data are presented as mean ± SD, ***P < 0.001 (unpaired, two-tailed Student's t-test). (C) Quantification of organoid diameter at day 7 of culture in control and knockout groups. Data are presented as mean ± SD, ***P < 0.001 (unpaired, two-tailed Student's t-test). (D) RT-qPCR analysis of Ki67 expression in control and ALKBH5-knockout organoids. Data are presented as mean ± SD, ***P < 0.01 (unpaired, two-tailed Student's t-test). (E) Bright-field images of human alveolar organoids cultured for 7 days with vehicle control (DMSO) or the ALKBH5 inhibitor 2,4-PDCA, together with quantification of the corresponding organoid diameters; scale bar, 100 µm. Organoid diameter was quantified from a total of 100 organoids, with each data point representing an individual organoid. Data are presented as mean ± SD, ***P < 0.001 (unpaired, two-tailed Student's t-test). (F) Immunofluorescence staining of Ki67 in control and ALKBH5-knockout organoids, together with quantification of Ki67^+^ cell proportions. Scale bar, 100 μm. Data are presented as mean ± SD, *P < 0.05 (unpaired, two-tailed Student's t-test). (G) Immunofluorescence staining showing expression of the AT1 marker AGER and the transitional-state marker KRT8 in control and ALKBH5-knockout organoids, with quantification of marker-positive cell fractions. Scale bar, 100 μm. Data are presented as mean ± SD, *P < 0.05 (unpaired, two-tailed Student's t-test). (H) RT-qPCR analysis of AT1 lineage markers AGER, AQP5, and T1α in control and knockout organoids. Data are presented as mean ± SD, *P < 0.05 (unpaired, two-tailed Student's t-test). (I) RT-qPCR analysis of AREG expression in control and ALKBH5-knockout organoids. Data are presented as mean ± SD, *P < 0.05 (unpaired, two-tailed Student's t-test).

To extend our findings to the human system, we employed human alveolar organoids derived from embryonic stem cells (ESCs) using a previously established protocol developed in our laboratory^33^. Human ESC-derived alveolar organoids expressed canonical AT2 markers, including SFTPC, LAMP3 and HTII-280 supporting their alveolar epithelial identity (Fig. S6). To knockout ALKBH5, we introduced two high-efficiency gRNAs targeting ALKBH5 via the PiggyBac (PB) transposon system^43^, while the control group received gRNAs targeting GFP. Doxycycline (Dox) induction triggered Cas9 expression, enabling precise genome editing. Following selection and validation, we successfully generated ALKBH5-deficient human alveolar organoids (Fig. 6A), confirmed by a marked reduction in ALKBH5 mRNA levels (Fig. 6B). Morphologically, ALKBH5-knockout organoids displayed significantly enlarged diameters compared to controls (Fig. 6C), indicating enhanced growth capacity. Immunofluorescence staining and qRT-PCR analyses of the proliferation marker Ki67 further demonstrated increased proliferative activity in ALKBH5-deficient organoids (Fig. 6D-E). Consistent with the genetic loss-of-function results, pharmacological inhibition of ALKBH5 demethylase activity using 2,4-PDCA^44^, ^45^, also led to a robust increase in organoid size (Fig. 6E), phenocopying the ALKBH5 knockout phenotype. At the concentration used, no apparent cytotoxicity was observed, as organoids maintained normal morphology and structural integrity, suggesting that the observed effects were not due to nonspecific toxicity. We next investigated the impact of ALKBH5 deletion on epithelial proliferation and differentiation. ALKBH5-deficient organoids exhibited increased proliferative activity, as indicated by Ki67 staining (Fig. 6F). Immunofluorescence staining further revealed elevated expression of AT1 markers and transitional-state markers in ALKBH5-deficient organoids (Fig. 6G). qRT-PCR analysis further confirmed significant upregulation of AT1 signature genes (Fig. 6H), indicating accelerated differentiation toward the AT1 lineage. Notably, ALKBH5-deficient organoids also exhibited increased AREG expression (Fig. 6I), paralleling our findings in the mouse system and suggesting a conserved regulatory mechanism. These results demonstrate that ALKBH5 constrains the growth and differentiation potential of human alveolar epithelial cells, in part through modulation of AREG expression, and validate the conserved role of m^6^A demethylation in human alveolar regeneration.

## Discussion

Alveolar regeneration requires precise control of AT2 cell activation and lineage progression, yet the upstream mechanisms coordinating these events remain poorly understood^2^, ^46^-^48^. Here, we identify Alkbh5 as a previously unrecognized suppressor of AT2-mediated regeneration, revealing a new functional role for this m⁶A RNA demethylase beyond its known functions in other stem cell systems^42^, ^49^, ^50^. While m⁶A modification has been shown to restrict stem cell expansion in embryonic stem cells^50^, hematopoietic stem cells^51^, and bone marrow mesenchymal stem cells^52^, its role in AT2 lineage dynamics has remained largely unexplored. We demonstrate that AT2-specific deletion of Alkbh5 markedly enhances AT2 proliferation and accelerates the AT2-to-AT1 transition across four mechanistically distinct injury models. This regenerative phenotype contrasts with reports in other tissues where loss of m⁶A demethylation can impair differentiation, highlighting that Alkbh5 functions in a lineage- and context-dependent manner. Our findings therefore broaden the functional repertoire of m⁶A demethylases in epithelial biology and position Alkbh5 as a critical brake that prevents premature or excessive AT2 activation under injury conditions. In addition, IGF2BP1/3 may function as m⁶A readers that stabilize Areg mRNA. While our data support this model, direct evidence of m⁶A-dependent binding will require future studies using approaches such as RIP or CLIP-based assays. Future studies will be needed to determine the precise binding sites and m⁶A dependency of IGF2BP1/3-Areg interactions at transcriptome-wide resolution.

Areg emerged as a key mechanistic effector linking Alkbh5 deletion to accelerated epithelial repair. Areg is known to promote regeneration across multiple organ injury contexts^53^-^55^, including the nervous system, gastrointestinal tissues, and hematopoietic compartments, underscoring its broad physiological relevance. Previous studies have emphasized immune-derived Areg—particularly from Tregs—as an important paracrine factor that facilitates epithelial repair by modulating stromal elements^31^. Our work identifies AT2/PATS cells themselves as a potent epithelial source of Areg after injury, and simultaneously reveals AT2 cells as a principal target of Areg-EGFR signaling. This dual identity—as both producer and responder—establishes an epithelial-intrinsic autocrine/paracrine loop that rapidly amplifies regenerative signals following injury. Our data clarify the context-dependent consequences of Areg activity: transient Areg induction accelerates epithelial regeneration, whereas sustained or spatially unrestricted Areg-EGFR activation, as exemplified by Cdc42-deficient models^40^, ^56^, can drive fibroblast activation and promote fibrotic progression. Thus, the timing and duration of Areg signaling dictate whether the outcome is regenerative or pathological. Importantly, our temporal analysis reveals dynamic changes in Alkbh5 and Areg expression during lung repair. Areg levels are elevated at early to intermediate stages, coinciding with enhanced AT2 proliferation and expansion of transitional PATS cells, and decline at later stages alongside recovery of Alkbh5 expression. This reciprocal pattern suggests a temporally coordinated regulation of the Alkbh5-Areg axis during alveolar regeneration. These findings highlight the context-dependent nature of Areg-EGFR signaling, in which transient activation promotes regeneration, whereas sustained activation may contribute to pathological remodeling and fibrosis.

Our human alveolar organoid experiments further reinforce that the Alkbh5-Areg-EGFR axis is evolutionarily conserved. Genetic ablation or pharmacological inhibition of ALKBH5 increased organoid growth, promoted AT1 differentiation, and elevated AREG expression, closely recapitulating the murine phenotypes. These findings indicate that epitranscriptomic control of AREG signaling represents a fundamental regulatory mechanism in human alveolar epithelial cells. Human alveolar organoids provide a physiologically relevant and experimentally tractable platform for modeling epithelial injury, repair, and lineage dynamics^57^-^60^. Given their ability to recapitulate key features of alveolar architecture and regenerative responses, these models offer valuable opportunities for dissecting human-specific regulatory circuits. The conserved response to ALKBH5 perturbation further underscores the utility of organoid-based systems for studying human lung regenerative biology and for informing future investigations into epithelial repair mechanisms.

These findings highlight the potential of ALKBH5 as a therapeutic target for lung repair. However, given its broad expression in lung tissues, systemic inhibition may lead to off-target effects. Therefore, cell type-specific targeting of AT2 cells is likely essential for safe therapeutic application. The accessibility of the lung via airway delivery offers a unique opportunity for localized intervention, which may reduce systemic exposure. These include viral vector-based systems^61^, ^62^ (e.g., AAV serotypes with tropism for alveolar epithelium), lipid nanoparticle (LNP)-mediated delivery of RNA^63^, small-molecule inhibitors and CRISPR-based gene modulation platforms^64^, all of which have shown potential for AT2-targeted gene regulation. Overall, selective and temporally controlled modulation of ALKBH5 in AT2 cells may represent a promising therapeutic direction, although further studies are needed to evaluate safety and long-term effects *in vivo*.

In summary, our study reveals an epithelial-intrinsic epitranscriptomic circuit in which Alkbh5 constrains AT2 activation by regulating the m⁶A-dependent stability of Areg mRNA. By showing consistent phenotypes across multiple injury paradigms and human organoid models, we establish Alkbh5 as a key suppressor of alveolar epithelial regeneration. These findings not only expand our understanding of how RNA modifications shape epithelial lineage behavior but also highlight the therapeutic potential of modulating the Alkbh5-Areg-EGFR axis to enhance lung repair.

## Supplementary Material

Supplementary figures and tables.

## Figures and Tables

**Figure 7:**
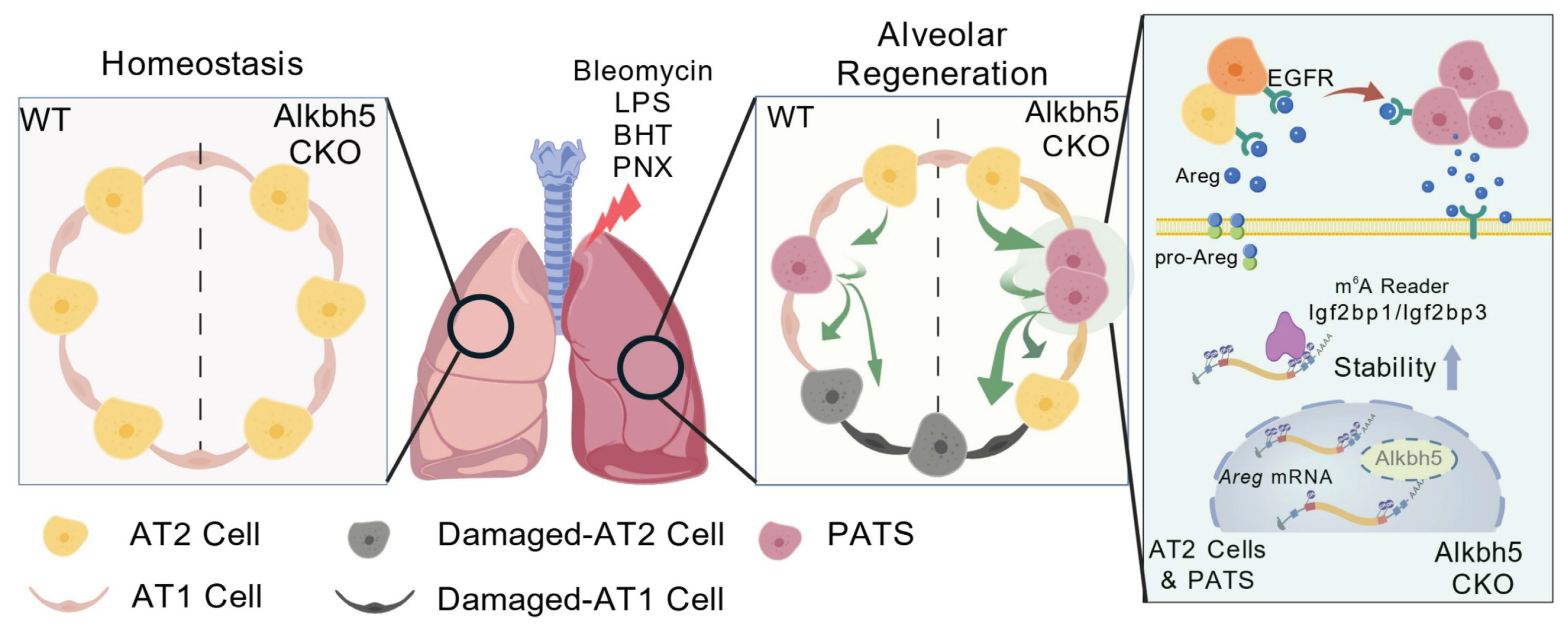
** Schematic summary of the functional and mechanistic consequences of AT2-specific Alkbh5 deletion.** Long-term lineage tracing demonstrates that AT2-targeted Alkbh5 deletion does not disrupt alveolar homeostasis under steady-state conditions. Following injury, Alkbh5-deficient lungs exhibit improved outcomes across multiple models, including reduced bleomycin-induced fibrosis, attenuated LPS- or BHT-mediated epithelial injury, and accelerated alveolar regrowth after PNX, attributable to enhanced AT2 proliferation and AT2→AT1 differentiation. At the molecular level, loss of Alkbh5 results in increased m⁶A deposition on Areg mRNA, which is selectively recognized by IGF2BP1/3, leading to enhanced Areg transcript stability. Upon epithelial injury, stabilized Areg is abundantly secreted by AT2 and transitional PATS cells, activating EGFR signaling. EGFR activation drives AT2 cells into a regenerative transitional state and reinforces Areg secretion, forming a positive-feedback loop that accelerates alveolar epithelial repair. Created with BioGDP.com^65^.

## Data Availability

All data supporting the findings of this study are available within the main text and Supplementary Materials. The scRNA-seq, RNA-seq, and MeRIP-seq datasets generated in this study have been deposited in the Gene Expression Omnibus (GEO) under the accession numbers GSE312245 (RNA-seq), GSE312382 (MeRIP-seq), and GSE312383 (scRNA-seq).
