# Supplementary material for: Loss of Alkbh5 enhances AT2 cell differentiation and alveolar repair across diverse injury models via m6A-dependent Areg signaling
Source: Int J Biol Sci. 2026 Jun 25;22(12):6465–82. doi: 10.7150/ijbs.130452 (PMC13411827; doi:10.7150/ijbs.130452)
Supplement: Supplementary file 1 — Supplementary figures and tables. [file ijbsv22p6465s1.pdf]

Supplementary Figures

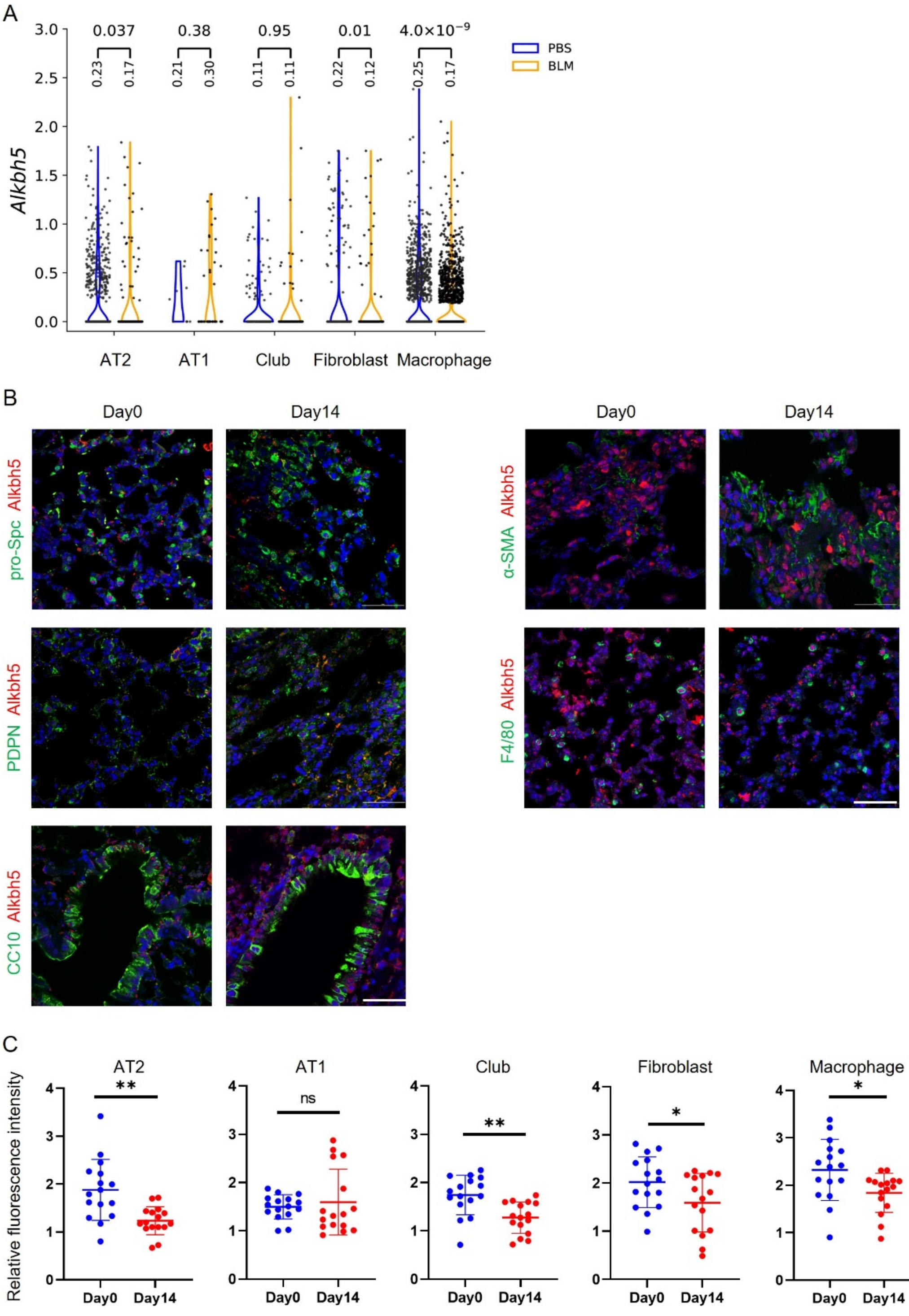

Figure S1 Cell type-specific expression of Alkbh5 in the lung under homeostatic and injury conditions

(A) Violin plots showing Alkbh5 expression across major lung cell populations under PBS (blue) and bleomycin (BLM, orange) conditions based on scRNA-

seq analysis. P values are indicated above each comparison. (B) Representative immunofluorescence images showing co-localization of Alkbh5 (red) with cell type–specific markers in lung tissue sections. AT2 cells were labeled with pro-SPC, AT1 cells with PDPN, club cells with CC10, mesenchymal cells with α-SMA, and macrophages with F4/80 (all in green). Nuclei were counterstained with DAPI (blue). Scale bars, 50μm. (C) Quantification of Alkbh5 fluorescence intensity in different cell populations under PBS and BLM conditions. Data are presented as mean ± SD from n = 4 mice per group. For each mouse, 4 randomly selected fields were analyzed. Each dot represents an individual field. Statistical analysis was performed across all fields. Data are presented as mean ± SD. * $p<0.05$, ** $p<0.01$, *** $p<0.001$ (unpaired, two-tailed Student's t-test).

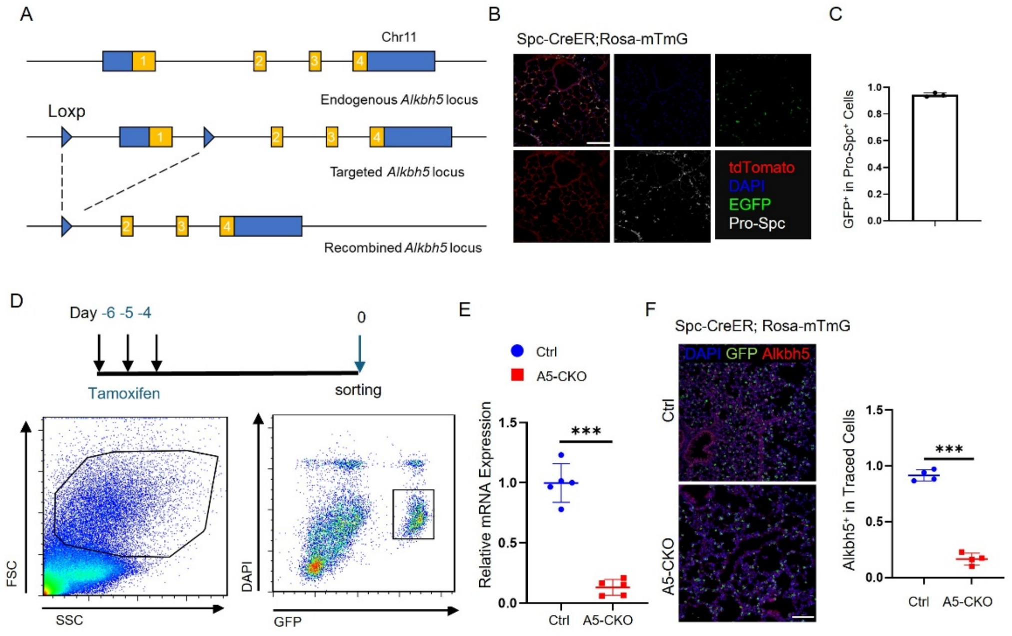

Figure S2 Generation and validation of conditional Alkbh5 knockout mice in AT2 Cells

(A) Schematic diagram of the Alkbh5 conditional knockout strategy. Loxp sites were inserted flanking the ATG and Exon 1 regions of the Alkbh5 gene; Cre-mediated recombination deletes Alkbh5. (B) Immunofluorescence staining of pro-SPC (AT2 cell marker) in lungs from Sftpc-CreERT2; Rosa26-mTmG mice following tamoxifen induction. Scale bars, 200μm. (C) Quantification of GFP^{+} AT2 cells from (B), data represent three mice. (D) Flow cytometric analysis of GFP-labeled AT2 cells in single-cell suspensions from tamoxifen-treated Sftpc-CreERT2; Rosa26-mTmG mice. (E) qPCR analysis of Alkbh5 mRNA expression in FACS-sorted AT2 cells. *** p <0.001 (unpaired, two-tailed Student's t-test). (F) Immunofluorescence staining of Alkbh5 protein in lung sections from control and Alkbh5 knockout mice, with quantification of staining intensity (n = 4 mice). Scale bar, 100 μm. *** p <0.001 (unpaired, two-tailed Student's t-test).

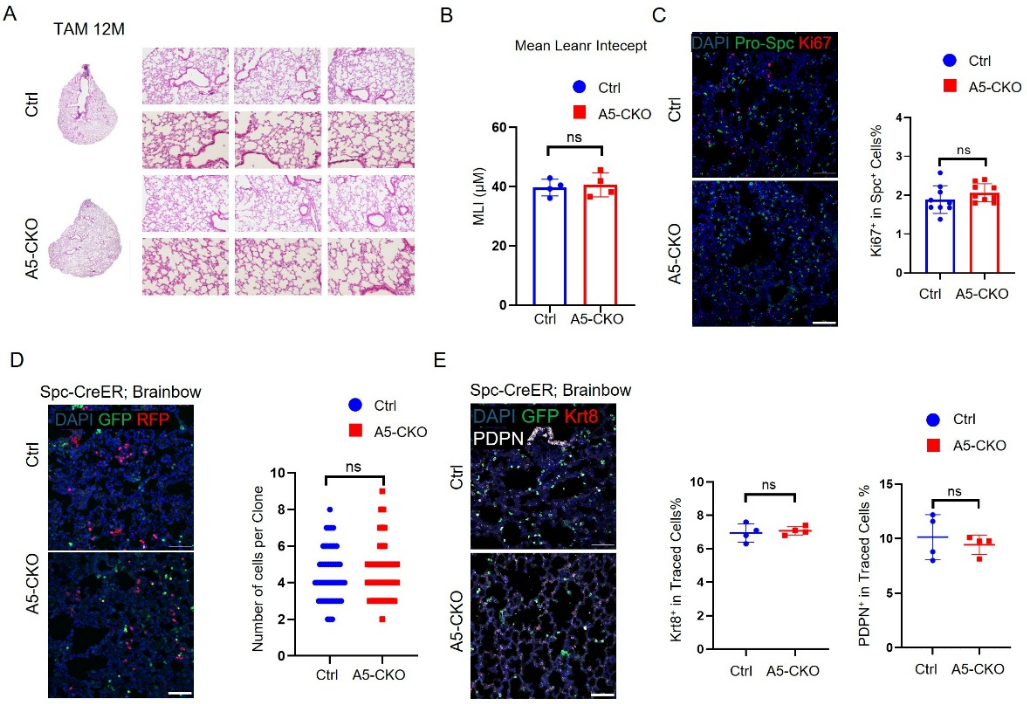

Figure S3 Knockout of Alkbh5 in AT2 cells does not affect the homeostasis of the lungs

(A) H&E staining of lung sections from control and Alkbh5 knockout mice one year after tamoxifen induction, showing gross morphology at low (4×) and medium (10×) magnifications. (B) Quantification of mean alveolar size based on six sections from four mice. Data are presented as mean ± SD, nonsignificant (NS) (unpaired, two-tailed Student's t-test). (C) Immunofluorescence staining of DAPI (blue), GFP (green), and Ki67 (red) in lung sections, with quantification of proliferating AT2 cells; data represent four mice. Scale bars, 100μm. Data are presented as mean ± SD. nonsignificant (NS) (unpaired, two-tailed Student's t-test). (D) Representative fluorescence images from Rosa26-Brainbow reporter mice showing RFP and GFP-labeled clones in control and Alkbh5-deficient lungs, with quantification of the number of cells per clone; data represent four mice. Data are presented as mean ± SD, nonsignificant (NS) (unpaired, two-tailed Student's t-test). (E) Immunofluorescence staining of DAPI, GFP, Krt8, and PDPN in control and Alkbh5-deficient lungs, with quantification of transitional ($Krt8^{+}$) and differentiated ($PDPN^{+}$) cells; data represent four mice. Scale bar, 100 μm. Data are presented as mean ± SD, nonsignificant (NS) (unpaired, two-tailed Student's t-test).

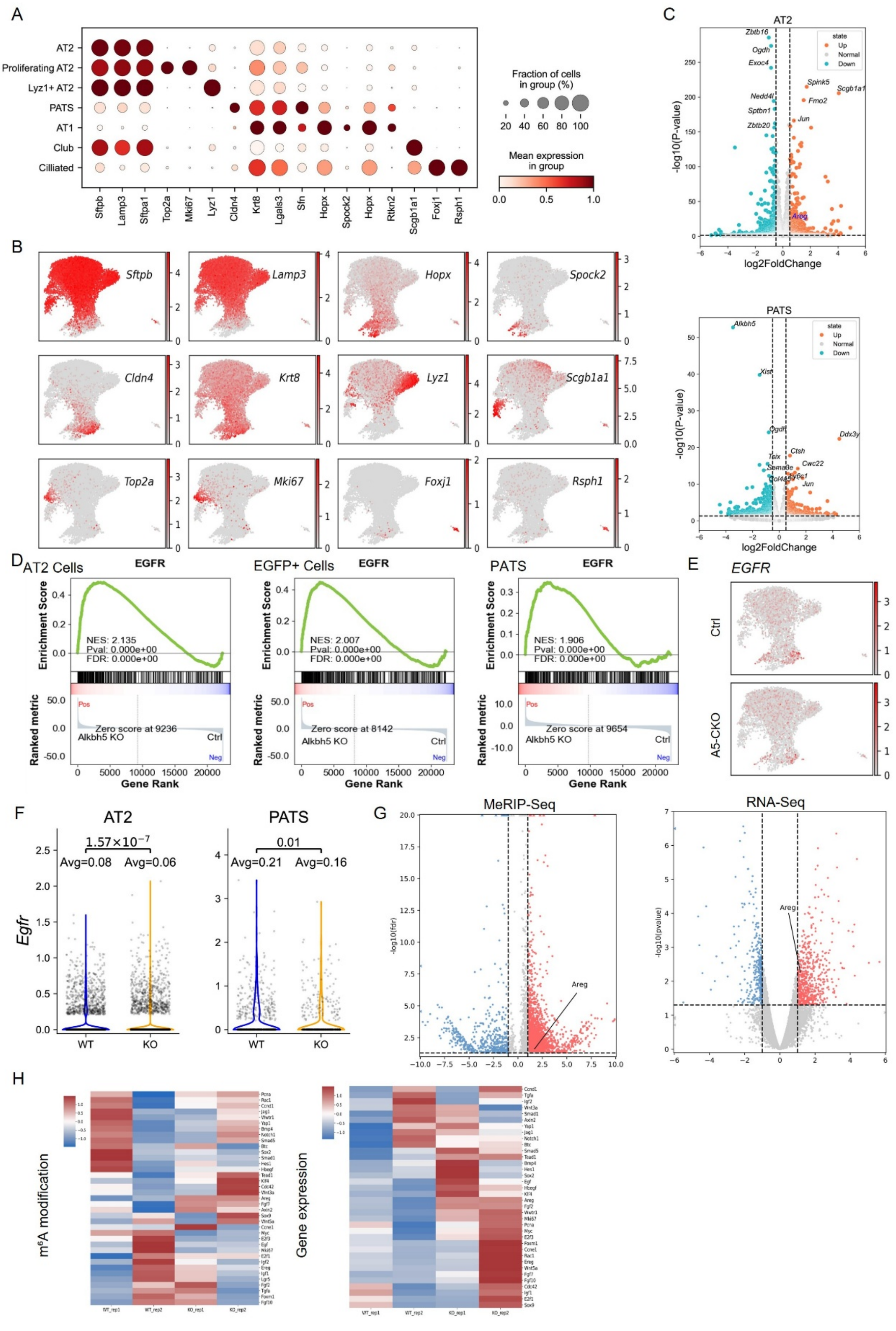

Figure S4 Integrated analysis of scRNA-seq, RNA-seq, and MeRIP-seq of AT2 cells following bleomycin-induced lung injury.

(A) Heatmap showing the expression of canonical lineage markers across EGFP⁺ epithelial populations isolated 14 days after bleomycin injury.m(B)

UMAP feature plots displaying representative marker genes used to define major epithelial subclusters. (C) Differential gene expression analysis of AT2 and pre-alveolar type I transitional state (PATS) cells. (D) EGFR pathway activity scores mapped across AT2, PATS, and AT1 cell populations. (E) UMAP plot showing the distribution of Egfr expression within $EGFP^{+}$ epithelial cells. (F) Egfr expression levels in AT2 and PATS subsets. (G) Integrated MeRIP-seq and RNA-seq tracks for Areg, showing increased m^6A peak enrichment and elevated transcript abundance in Alkbh5-deficient AT2 cells. (H) Heatmap showing differential m^6A modification (MeRIP-seq) and (RNA-seq) of regeneration-associated genes in WT and Alkbh5-deficient AT2 cells.

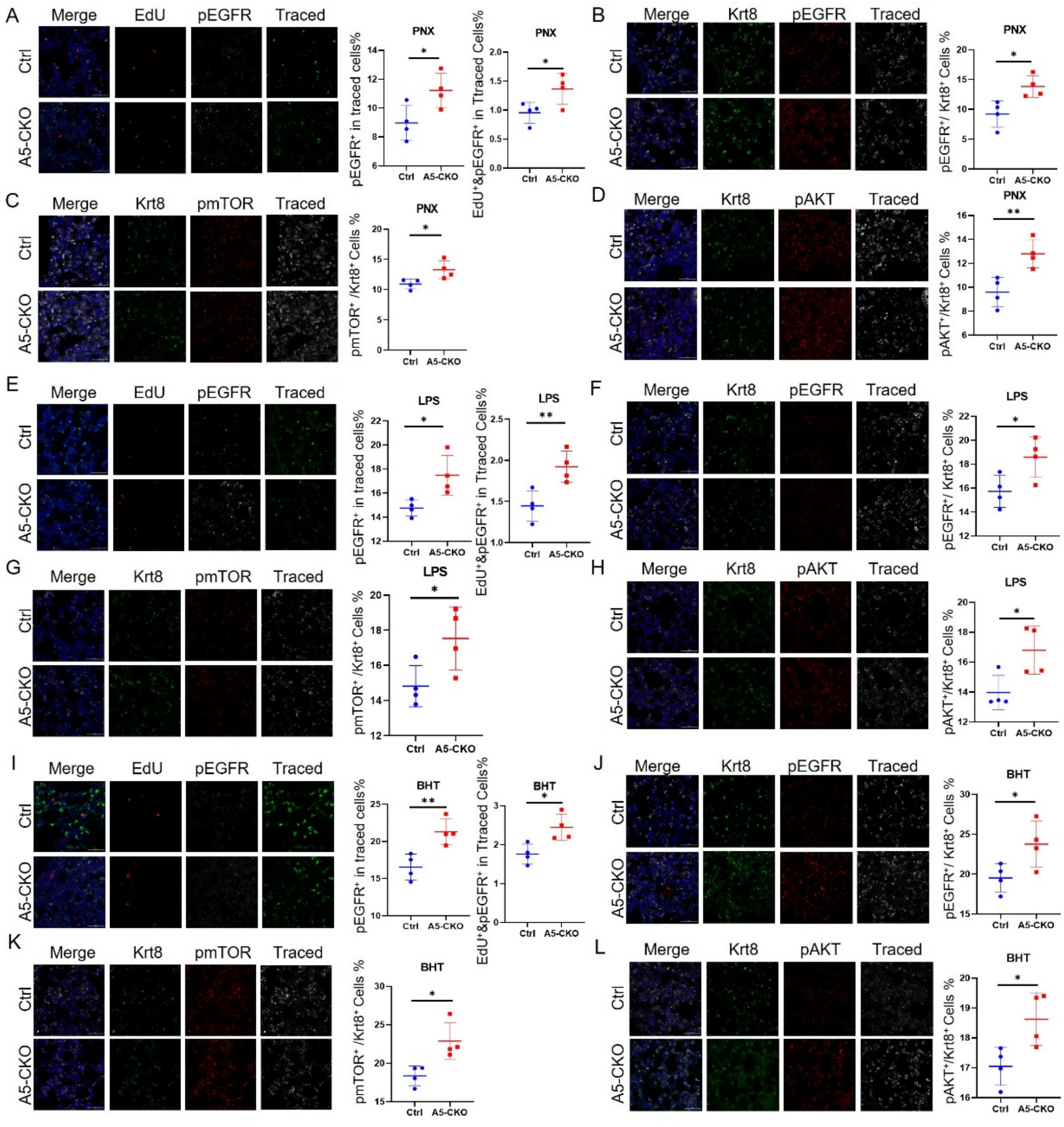

Figure S5 Activation of EGFR signaling in AT2 cells across PNX, LPS, and BHT-induced lung injury models

(A) Immunofluorescence staining of lung sections after PNX. EdU (red) labels proliferating cells, p-EGFR (white) marks EGFR activation, and Traced (green) indicates lineage-labeled AT2 cells. (B–D) Immunofluorescence staining and quantification of phosphorylated EGFR (p-EGFR, red), mTOR (p-mTOR, red), and AKT (p-AKT, red) in transitional AT2 cells (Krt8$^+$, green) following PNX. Traced (white) marks lineage-labeled cells. (E) Immunofluorescence staining of lung sections after LPS-induced injury. EdU (red) labels proliferating cells, p-EGFR (white) marks EGFR activation, and Traced (green) indicates lineage-labeled AT2 cells. (F–H) Immunofluorescence staining and quantification of phosphorylated EGFR (p-EGFR, red), mTOR (p-mTOR, red), and AKT (p-AKT,

red) in transitional AT2 cells (Krt8$^+$, green) after LPS-induced injury. Traced (white) indicates lineage-traced cells. (I) Immunofluorescence staining of lung sections after BHT-induced injury. EdU (red) labels proliferating cells, p-EGFR (white) marks EGFR activation, and Traced (green) indicates lineage-labeled AT2 cells. (J–L) Immunofluorescence staining and quantification of phosphorylated EGFR (p-EGFR, red), mTOR (p-mTOR, red), and AKT (p-AKT, red) in transitional AT2 cells (Krt8$^+$, green) after BHT-induced lung injury. Traced (white) marks lineage-labeled AT2 cells. Scale bars, 50 μm. All values are presented as mean ± SD; *$p < 0.05$, **$p < 0.01$ (unpaired, two-tailed Student's t-test).

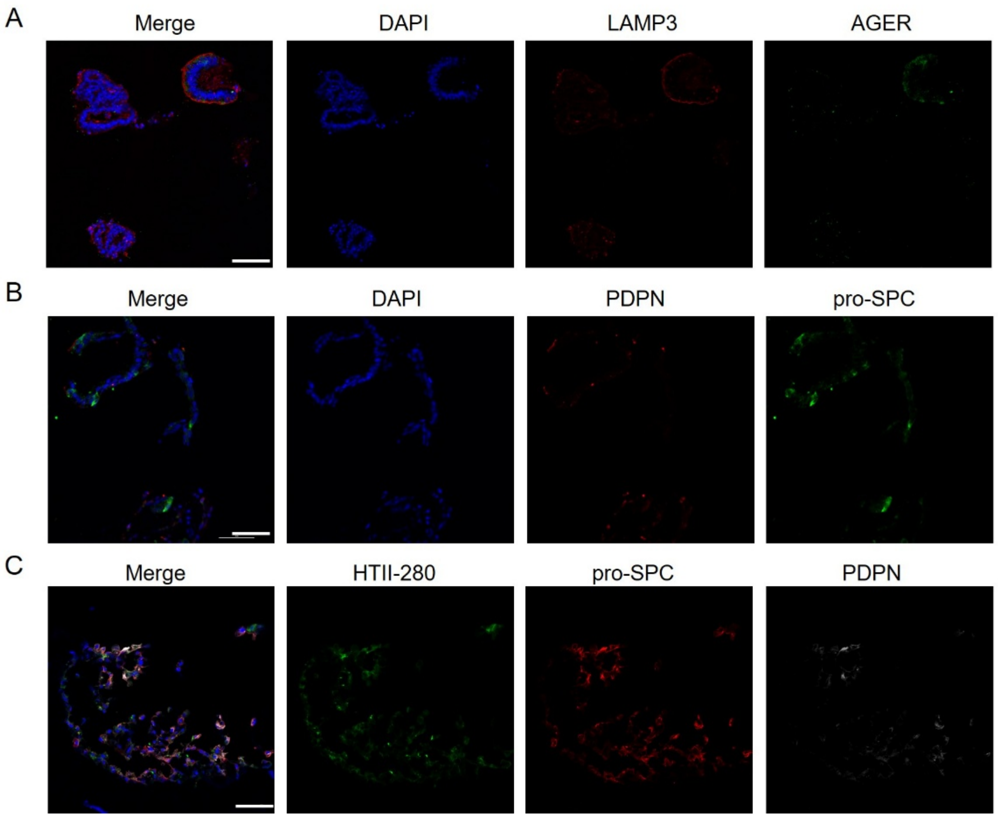

Figure S6 Characterization of human ESC-derived alveolar organoids

(A) Immunofluorescence staining of AT2 marker LAMP3 and AT1 marker AGER in alveolar organoids. (B) Immunofluorescence staining of AT2 marker pro-SPC and AT1 marker PDPN. (C) Immunofluorescence staining of AT2 markers HTII-280 and pro-SPC, together with AT1 marker PDPN, in alveolar organoids. Scale bars, 100 μm.

Table 1 Chemicals, peptides, and recombinant proteins

Bleomycin	TCI	Cat#: B3972
Butylated hydroxytoluene (BHT)	Sigma	Cat#: B1378
LPS	Sigma	Cat#: L2360
EGF	MCE	Cat#: HY-P7067
ROCK-Inhibitor (Y-27632)	MCE	Cat#: HY-U00351
CHIR 99021	Tocris	Cat#: 4423
Dexamethasone	Sigma	Cat#: D4902
human FGF10	R&D Systems,	Cat#: 345-FG-025
human KGF	Novoprotein	Cat#: CM88
ITS premix	Corning	Cat#: 354351
DAPT	Sigma	Cat#: D5942
8-Br-cAMP	Biolog Life Science Institute	Cat#: B007-500
3-isobutyl-1-methylxanthine	Wako	Cat#: 09503413
ITS premix	Corning	Cat#: 354351
SB431542	MCE	Cat#: HY-10431
mouse Amphiregulin	MCE	Cat#: HY-P77868
mouse Noggin	Novoprotein	Cat#: C028
mouse Fgf7	MCE	Cat#: HY-P7176
Insulin	GLPBIO	Cat#: GC31303
Transferrin	Sigma	Cat#: T8158
Cholera toxin	Sigma	Cat#: C8052
Epidermal Growth Factor	Sigma	Cat#: E4127
Bovine Pituitary Extract	MACGENE	Cat#: CC203
Retinoic acid	Sigma	Cat#: R2625
Tamoxifen	MCE	Cat#: HY-13757A
Actinomycin D	GLPBIO	Cat#: GC16866
Matrigel Matrix	Corning	Cat#: 356237
Dispase II	Roche	Cat#: 4942078001
Collagenase, Type I	MCE	Cat#: HY-E70005A
DAPI	Thermo Fisher Scientific	Cat#: D1306
2,4-PDCA	MCE	Cat#: HY-W017132
Penicillin-Streptomycin Mixed Solution	Gibco	Cat#: 15140-122
RBC Lysis Buffer (10X)	eBioscience	Cat#: 00-4333-57
Dnase I	Solarbio	Cat#: D8071
HiScript II Q RT SuperMix for qPCR	Vazyme	Cat#: R223-01
Taq Pro Universal SYBR qPCR Master Mix	Vazyme	Cat#: Q712-03
Trypsin-EDTA (0.25%), phenol red	Thermo Fisher Scientific	Cat#: 25200056
Magnetic protein G beads	Thermo Fisher Scientific	Cat#: 10004D
RNA Fragmentation Reagents	New England Biolabs	Cat#: E6150S
DPBS	Gibco	Cat#: C14190500BT
GlutaMAX Supplement	Gibco	Cat#: 35050061
N6-Methyladenosine Enrichment Kit	New England Biolabs	Cat#: E1610S

Table 2 Antibodies list

REAGENT or RESOURCE	DILUTION	SOURCE	IDENTIFIER
Rat anti-Ki67	1:200	Thermo Fisher Scientific	Cat#: 14-5698-82
Rat anti-Krt8	1:200	DSHB	Cat#: TROMA-I
Rabbit anti-Alkbh5	1:100	Proteintech	Cat#:16837-1-AP
Mouse anti- Alkbh5	1:100	Proteintech	Cat#: 67811-1-Ig
Mouse anti-HT2-280	1:100	Terrace Biotech	Cat#: TB-27AHT2-280
Rabbit anti-CC10	1:200	Abcam	Cat#: ab40873
Rabbit anti-GFP	1:200	MCE	Cat#: HYP80141
Goat anti-GFP	1:200	Abcam	Cat#: ab6673
Rabbit anti-pro-SPC	1:200	Millipore	Cat#: AB3786
Rabbit anti-AQP5	1:200	Abcam	Cat#: AB92320
Rabbit anti-α-SMA	1:200	Proteintech	Cat#: 14395-1-AP
Goat anti-PDPN	1:50	R&D Systems	Cat#: AF3244
Goat anti-Areg	1:200<br>50ng/ml in Cell Culture	R&D Systems	Cat#: AF989
IgG (Goat)	50ng/ml in Cell Culture	R&D Systems	Cat#: AB108C
Rat anti-Ki67	1:200	Thermo Fisher Scientific	Cat#: 14-5698-82
Rabbit anti-Phospho-EGFR (Tyr845)	1:200	Cell Signaling Technology	Cat#: 2231
Rabbit anti-Phospho-Akt (Ser473)	1:200	Cell Signaling Technology	Cat#: 4060
Rabbit anti-Phospho-mTOR (Ser2448)	1:200	Cell Signaling Technology	Cat#: 5536
Mouse anti-LAMP3	1:200	Diaclone	Cat#: 857.770.000
Rat anti-F4/80	1:200	Abcam	Cat#: ab90247
Alexa Fluor 488 Donkey anti-rabbit	1:500	Life Technology	Cat#: A-21206
Alexa Fluor 568 Donkey anti-rabbit	1:500	Life Technology	Cat#: A10042
Alexa Fluor 647 Donkey anti-rabbit	1:500	Life Technology	Cat#: A-31573
Alexa Fluor 488 Donkey anti-mouse	1:500	Life Technology	Cat#: A-21202
Alexa Fluor 647 Donkey anti-mouse	1:500	Life Technology	Cat#: A-31571
Alexa Fluor 568 Donkey anti-goat	1:500	Life Technology	Cat#: A-11057
Alexa Fluor 647 Donkey anti-rat	1:500	Life Technology	Cat#: A48272

Table 3 RT-qPCR primer list

Gene	Forward primer (5' to 3')	Reverse primer (5' to 3')
mGAPDH	AATGGATTTGGACGCATTGGT	TTTGCACTGGTACGTGTTGAT
mAlkbh5	TCGGAACCTGTGCTTTCTCTGC	CTTCCTGAGAATGATGACCGCC
mAQP5	TCCATGAACCCAGCCCGATCTT	GAAGTAGAGGATTGCAGCCAGG
mAger	GCCACTGGAATTGTCGATGAGG	GCTGTGAGTTCAGAGGCAGGAT
mPDPN	ACAACCACAGGTGCTACTGGAG	GTTGCTGAGGTGGACAGTTCCT
mHopx	TCTCCATCCTTAGTCAGACGC	GGGTGCTTGTTGACCTTGTT
mT1α	ACAGGTGCTACTGGAGGGCTT	TCCTCTAAGGGAGGCTTCGTC
mIg2bp1	CCTGGCTCATAACAACTTCGTCG	CCTTCACAGTGATGGTCCTCTC
mIg2bp2	TGAAGCCTGTGCCAATGCTGAG	CCAGTCGAAAAGATGCCAAGTGC
mIg2bp3	CCACCCAGTTTGTTGGAGCCAT	GGATAGTAATGGACTTCTCCGCG
hGAPDH	GGAGCGAGATCCCTCCAAAAT	GGCTGTTGTCATACTTCTCATGG
hALKBH5	CCAGCTATGCTTCAGATCGCCT	GGTTCTCTTCCTTGTCCATCTCC
hHOPX	ATTCCACCACGCTGTGCCTCAT	AGTCTGTGACGGATCTGCACTC
hAGER	GCCACTGGTGCTGAAGTGTA	TGGTCTCCTTTCCATTCCTG
hAQP5	TACGGTGTGGCACCGCTCAATG	AGTCAGTGGAGGCGAAGATGCA
Gene	Forward primer (5' to 3')	Reserve primer (5' to 3')
mGAPDH	AATGGATTTGGACGCATTGGT	TTTGCACTGGTACGTGTTGAT
mAlkbh5	TCGGAACCTGTGCTTTCTCTGC	CTTCCTGAGAATGATGACCGCC
mAQP5	TCCATGAACCCAGCCCGATCTT	GAAGTAGAGGATTGCAGCCAGG
mAger	GCCACTGGAATTGTCGATGAGG	GCCACTGGAATTGTCGATGAGG
mPDPN	ACAACCACAGGTGCTACTGGAG	ACAACCACAGGTGCTACTGGAG
mHopx	TCTCCATCCTTAGTCAGACGC	TCTCCATCCTTAGTCAGACGC
mT1α	ACAGGTGCTACTGGAGGGCTT	ACAGGTGCTACTGGAGGGCTT
mIgbp1	CCTGGCTCATAACAACTTCGTCG	CCTTCACAGTGATGGTCCTCTC
mIgbp2	TGAAGCCTGTGCCAATGCTGAG	CCAGTCGAAAAGATGCCAAGTGC
mIgbp3	CCACCCAGTTTGTTGGAGCCAT	GGATAGTAATGGACTTCTCCGCG
hGAPDH	GGAGCGAGATCCCTCCAAAAT	GGCTGTTGTCATACTTCTCATGG
hALKBH5	CCAGCTATGCTTCAGATCGCCT	GGTTCTCTTCCTTGTCCATCTCC
hHOPX	ATTCCACCACGCTGTGCCTCAT	AGTCTGTGACGGATCTGCACTC
hAGER	GCCACTGGTGCTGAAGTGTA	TGGTCTCCTTTCCATTCCTG
hAQP5	TACGGTGTGGCACCGCTCAATG	AGTCAGTGGAGGCGAAGATGCA
